# The genetic and functional analysis of flavor in commercial tomato: the *FLORAL4* gene underlies a QTL for floral aroma volatiles in tomato fruit

**DOI:** 10.1111/tpj.14795

**Published:** 2020-06-21

**Authors:** Yury M. Tikunov, Raana Roohanitaziani, Fien Meijer‐Dekens, Jos Molthoff, Joao Paulo, Richard Finkers, Iris Capel, Fatima Carvajal Moreno, Chris Maliepaard, Mariska Nijenhuis‐de Vries, Caroline W. Labrie, Wouter Verkerke, Adriaan W. van Heusden, Fred van Eeuwijk, Richard G. F. Visser, Arnaud G. Bovy

**Affiliations:** ^1^ Plant Breeding Wageningen University and Research Droevendaalsesteeg 1 Wageningen 6708PB the Netherlands; ^2^ Biometris Wageningen University and Research Droevendaalsesteeg 1 Wageningen 6708PB the Netherlands; ^3^ Food & Biobased Research Wageningen University and Research Bornse Weilanden 9 Wageningen 6708WG the Netherlands; ^4^ Greenhouse Horticulture Wageningen University and Research Violierenweg 1 Bleiswijk 2665MV the Netherlands

**Keywords:** tomato, *Solanum lycopersicum*, aroma, flavor, quantitative trait loci, volatiles, 2‐phenylethanol

## Abstract

Tomato (*Solanum lycopersicum* L.) has become a popular model for genetic studies of fruit flavor in the last two decades. In this article we present a study of tomato fruit flavor, including an analysis of the genetic, metabolic and sensorial variation of a collection of contemporary commercial glasshouse tomato cultivars, followed by a validation of the associations found by quantitative trait locus (QTL) analysis of representative biparental segregating populations. This led to the identification of the major sensorial and chemical components determining fruit flavor variation and detection of the underlying QTLs. The high representation of QTL haplotypes in the breeders’ germplasm suggests that there is great potential for applying these QTLs in current breeding programs aimed at improving tomato flavor. A QTL on chromosome 4 was found to affect the levels of the phenylalanine‐derived volatiles (PHEVs) 2‐phenylethanol, phenylacetaldehyde and 1‐nitro‐2‐phenylethane. Fruits of near‐isogenic lines contrasting for this locus and in the composition of PHEVs significantly differed in the perception of fruity and rose‐hip‐like aroma. The PHEV locus was fine mapped, which allowed for the identification of *FLORAL4* as a candidate gene for PHEV regulation. Using a gene‐editing‐based (CRISPR‐CAS9) reverse‐genetics approach, *FLORAL4* was demonstrated to be the key factor in this QTL affecting PHEV accumulation in tomato fruit.

## Significance Statement

In commercial tomato breeding, elite breeding lines remain the major source of new cultivars. We studied the sensorial, chemical and genetic components of fruit flavor in a collection of commercial glasshouse tomatoes. The haplotypes associated with the flavor QTLs characterized in this study were well represented in the current breeders’ germplasm. Fine mapping of the major QTLs for floral phenolic volatiles resulted in the identification and functional confirmation of the causal gene.

## INTRODUCTION

Tomato (*Solanum lycopersicum* L.) flavor is determined by a combination of taste, aroma and mouthfeel attributes. A combination of several sugars and organic and amino acids primarily determines the taste of tomato fruit, whereas a few dozen volatile organic compounds have been considered to actively contribute to the tomato fruit aroma (Buttery *et al*., 1987; Buttery *et al*., 1988; Baldwin *et al*., 1998; Krumbein and Auerswald, 1998; Baldwin *et al*., 2000b; Tandon *et al*., 2003; Kazeniac and Hall, 2006; Baldwin *et al*., 2008; Du *et al*., 2015). Despite their importance, much less is known of the fruit‐texture factors affecting mouthfeel characteristics. As a result of major advances in genomics resources over the past decade, such as a tomato reference genome sequence (The Tomato Genome Consortium, 2012) and the re‐sequencing data of hundreds of tomato accessions (Causse *et al*., 2013; Tomato Genome Sequencing Consortium *et al*., 2014; Tieman *et al*., 2017), tomato has become a model for genetic studies of fruit‐quality traits, and a number of studies have been performed to discover the genetic loci of the tomato genome that control the quantitative variation of flavor‐related chemicals (Tanksley *et al*., 1996; Fulton *et al*., 1997; Bernacchi *et al*., 1998; Frary *et al*., 2000; Causse *et al*., 2001; Saliba‐Colombani *et al*., 2001; Causse *et al*., 2002; Fulton *et al*., 2002; Tadmor *et al*., 2002; Mathieu *et al*., 2009; Prudent *et al*., 2009; Zanor *et al*., 2009; Bauchet *et al*., 2017; Tieman *et al*., 2017; Zhao *et al*., 2019). These genetic studies have adopted different complementary strategies regarding the material and the methodologies used. They often make use of genetically distant material, combining cultivated and wild tomato relatives or heirloom varieties, which maximizes genetic and phenotypic diversity and, therefore, increases the chances to find significant quantitative trait loci (QTLs). Although the genetic diversity of the contemporary commercial tomato germplasm remains relatively narrow, compared with wild tomato relatives, there has been a marked trend of diversification in both genetic and chemical flavor‐related diversity in recent decades (Blanca *et al*., 2015; Schouten *et al*., 2019). Therefore, a systematic analysis of the chemical, sensorial and genetic components of tomato fruit quality in the contemporary commercial tomato will facilitate the more efficient use of the available diversity, as well as the identification of gaps.

The specific molecular factors – genes and enzymes – underlying the effects of the flavor QTL regions largely remain unknown. The boundaries of genetic loci associated with fruit quality components may deviate from study to study, but often remain uncertain and large, and may therefore contain hundreds of genes, the annotation of which often remains unknown or tentative, and the number of functionally explored genes remains limited. A genetic marker tightly associated with a trait is already an effective tool to perform selection for a trait in practical breeding, particularly when the variation for a trait is determined by only one or a very limited number of genes. Flavor, however, is a highly complex multigenic trait, which develops as a product of the sensorial activity of many chemical compounds. These chemicals, in turn, are produced via different biosynthetic pathways consisting of multiple reactions mediated by different enzymes. The productivity of the biochemical reactions or pathways can be affected by different molecular, physiological and environmental factors, resulting in qualitative and quantitative changes in chemical composition of different magnitudes. For instance, natural mutations in genes encoding master regulators of tomato fruit ripening, such as *rin*, *nor* and *cnr*, affect multiple biochemical pathways (Kovács *et al*., 2009), whereas more downstream pathway‐specific transcription factors, such as *SlMYB12*, may affect only one or a few related metabolic pathways (Ballester *et al*., 2010). Specific structural genes, e.g. the non‐smoky glycosyltransferase *NSGT1*, may affect the concentration of one or a few specific chemicals under particular circumstances (Tikunov *et al*., 2013). Detailed knowledge of the nature of the molecular factor(s) underlying flavor traits may facilitate: (i) more targeted breeding for particular flavor components; (ii) understanding interactions and forecasting the performance of flavor QTLs; (iii) the discovery of natural genetic variants that provide a more favorable trait performance; or (iv) the creation of novel favorable variants using mutagenesis or gene‐editing approaches.

The aroma‐active volatiles of tomato fruit are classified into several distinct biochemical groups according to the common precursor they are derived from: amino acids, such as phenylalanine, leucine and isoleucine, fatty acids and carotenoids. One of the most important groups of volatiles that contribute to the aroma of tomato fruit are phenolic volatiles: phenylacetaldehyde, 2‐phenylethanol and 1‐nitro‐2‐phenylethane, which are directly catabolized from phenylalanine, and are often associated with sweet, fruity and floral aromas (Baldwin *et al*., 2000a, Tieman *et al*., 2007, Tzin *et al*., 2013, Rambla *et al*., 2014). A number of genetic loci responsible for quantitative variation in PHEVs have been described (Zanor *et al*., 2009; Bauchet *et al*., 2017; Tieman *et al*., 2017); however, the molecular factors and mechanisms underlying this variation in cultivated tomato remain unknown.

Here we present a study of the sensorial, chemical and genetic components of tomato fruit flavor, conducted systematically, beginning with an analysis of variation in a collection of commercial glasshouse tomato germplasm, followed by the discovery of the QTLs associated with the sensorial and chemical variation in segregating populations and an analysis of the distribution of the QTL haplotypes in the contemporary commercial tomato. One of the QTLs important for tomato fruit flavor quality was fine mapped to a region harboring only 10 genes. Reverse‐genetics analysis led to the discovery of a *FLORAL4* gene underlying the quantitative variation of phenolic volatiles in tomato fruit.

## RESULTS

### The main components of the genetic, chemical and sensorial variation

To assess chemical, sensorial and genetic diversity in cultivated tomato, a diversity panel (DP) of 94 cultivars was established by asking several leading breeding companies for cultivars of different genetic backgrounds and diverse fruit flavor characteristics. A comprehensive analysis of volatile compounds in fruits of this collection was performed and has been reported previously (Tikunov *et al*., 2005; Ursem *et al*., 2008; Menéndez *et al*., 2012). To study the association between the genetic, chemical and sensorial variation further, a half diallel was developed by crossing the parents of four cultivars representative of the variation across the panel. These four cultivars, here coded C074 and C085 (cherry fruits), and R075 and R104 (round fruits of intermediate size), were chosen as the best representatives of the genetic diversity of the entire DP. Among the cultivars of the corresponding fruit types these four had the highest total loading values across the top‐20 principal components (PCs) of the genetic principal component analysis (PCA), which altogether captured 85% of the total genetic variation (Figure S1). Fruits of six F_2_ and three F_6_ populations of the half diallel were genotyped and then analysed for chemical composition and sensory attributes, in multiple trials (Table S11).

**Figure 1:**
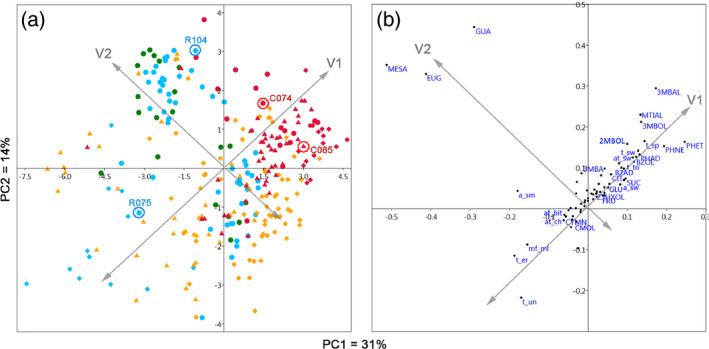
Principal component analysis of the sensory and metabolic profiles of the diversity panel (DP, circles), F_2_ (diamonds) and F_6_ (triangles) fruit material. (a) Variation structure of the fruit material along the first two major sources of the chemical and sensorial variation, displayed in plot (b). V1 and V2 are the two main vectors of variation drawn according to the visual clustering of the chemical and sensorial components. Color key: red, cherry tomatoes; blue, round cultivars (intermediate fruit size in DP); green, beef (large fruit size in DP); yellow, cherry × round progeny. The four cultivars selected to be parents of the crossing populations are encircled in (a). Chemical abbreviations: BZAD, benzaldehyde; BZOL, benzyl alcohol; CIT, citric acid; CMOL, p‐cymenol; CYMN, p‐cymene; EUG, eugenol; FRU, fructose; GLU, glucose; GUA, guaiacol; 3MBA, 3‐methylbutanoic acid; 3MBAL, 3‐methylbutanal; 2MBOL, 2‐methylbutanol; 3MBOL, 3‐methylbutanol; MESA, methyl salicylate; MTIAL, methional; PHAD, phenylacetaldehyde; PHET, 2‐phenylethanol; PHNE, 1‐nitro‐2‐phenylethane; SUC, sucrose; Z3HXOL, Z‐3‐hexanol. Taste abbreviations: a_sm, smoky aroma; a_sw, sweet aroma; at_bit, aftertaste bitter; at_ch, chemical aftertaste; t_er, taste earthy; t_to, taste tomato; t_sp, spicy taste; t_sw, sweet taste; t_un, unripe taste.

To determine the major variation components of the sensorial and chemical compositions of the tomato material studied, a PCA was performed on a total set of 273 sensorial and chemical profiles, obtained from DP, F_2_ and F_6_ fruit material. Two major vectors of the chemical and sensorial variation could be observed in the two‐dimensional space formed by the first two PCs, which captured 31 and 14% of the total variation, respectively (Figure 1). The first vector of the variation (V1; Figure 1) is determined by quantitative variation of several chemicals of different biochemical origins. These chemicals are derived from the catabolism of: (i) amino acids – 2‐phenylethanol (PHET), phenylacetaldehyde (PHAL), 1‐nitro‐2‐phenylethane (PHNE), 3‐methylbutanal (3MBAL), 2‐ and 3‐methylbutanol (2MBOL and 3MBOL) and methional (MTIAL); (ii) benzoic acid metabolites – benzaldehyde (BZAD) and benzyl alcohol (BZOL); and (iii) sugars – glucose (GLU), fructose (FRU) and sucrose (SUC) (Figure 1b). Sensory attributes of ‘taste tomato’, ‘taste spicy’ and all the three sweet attributes (‘sweet aroma’, ‘sweet taste’ and ‘sweet aftertaste’) also showed high loading values along this vector of variation, suggesting a positive correlation with the high content of the chemicals mentioned above, whereas unripe and earthy taste as well as mealy mouthfeel had low scores when high levels of these chemicals were present in fruits. Cherry‐type tomato fruits of both the DP and the cherry–cherry crossing populations had higher positive scores along the first vector of variation compared with the larger fruit types in the variation space formed by the first two principal components (Figure 1a). The cherry–round cross progenies seemed to share the chemical properties of both the parental types, as they spanned the entire variation space between the smaller and the larger fruit types. The second vector of variation (V2) was determined by variation in phenylpropanoid volatiles (PHPVs) guaiacol (GUA), eugenol (EUG) and methyl salicylate (MESA), and the sensory attribute ‘smoky aroma’, which separated all the fruit material into two distinct groups. The PHPV/‘smoky aroma’ contrast was less dependent on fruit type, as V2 is orthogonal to the cherry‐dependent V1, suggesting no correlation between the quantitative patterns of the chemicals and the sensory attributes determining both vectors (Figure 1a).

To estimate the statistical importance of individual chemical components for explaining the variation of sensorial characteristics, a random forest (RF) regression analysis was performed (Table 1). As also suggested by the PCA, the PHPVs eugenol, methyl salicylate and guaiacol had positive coefficients in the models for several unpleasant flavor characteristics: smoky aroma, bitter, chemical, rough aftertaste, and earthy and unripe tastes. On the other hand, PHPVs were negatively associated with the expression of sweet, tomato‐like and spicy flavors. Phenylalanine catabolites 2‐phenylethanol, phenylacetaldehyde, 1‐nitro‐2‐phenylethane and the leucine‐derived volatile organic compounds (VOCs), in particular 3‐methylbutanal, together with sugars and citric acid positively contributed to the models of more pleasant flavor characteristics, such as sweet taste, aftertaste and aroma. Compounds of other biochemical origins, such as the carotenoid‐derived geranylacetone and 6‐methyl‐5‐hepten‐2‐one were negatively associated with tomato‐like flavor. The amino‐acid‐derived methional and the terpenoids *p*‐cymenol and *m*‐cymene also had significant importance and a negative effect in the models of spicy and sweet sensory attributes.

**Table 1:**
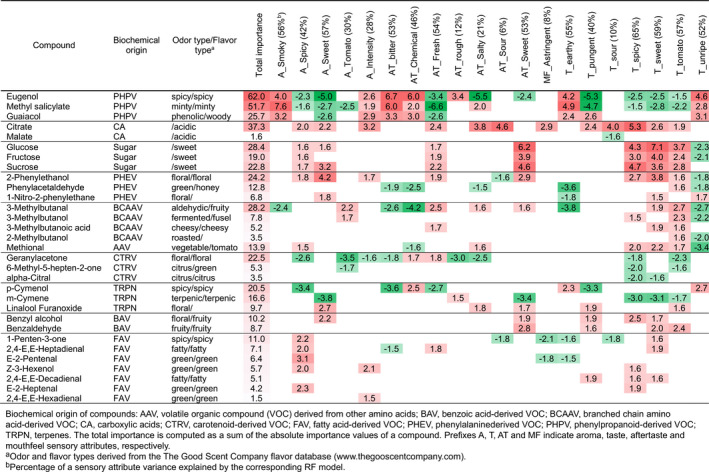
The importance of chemical compounds in the random forest regression (RFR) models of the sensory characteristics of tomato fruit.

### Flavor QTLs of commercial tomato

**Figure 2:**
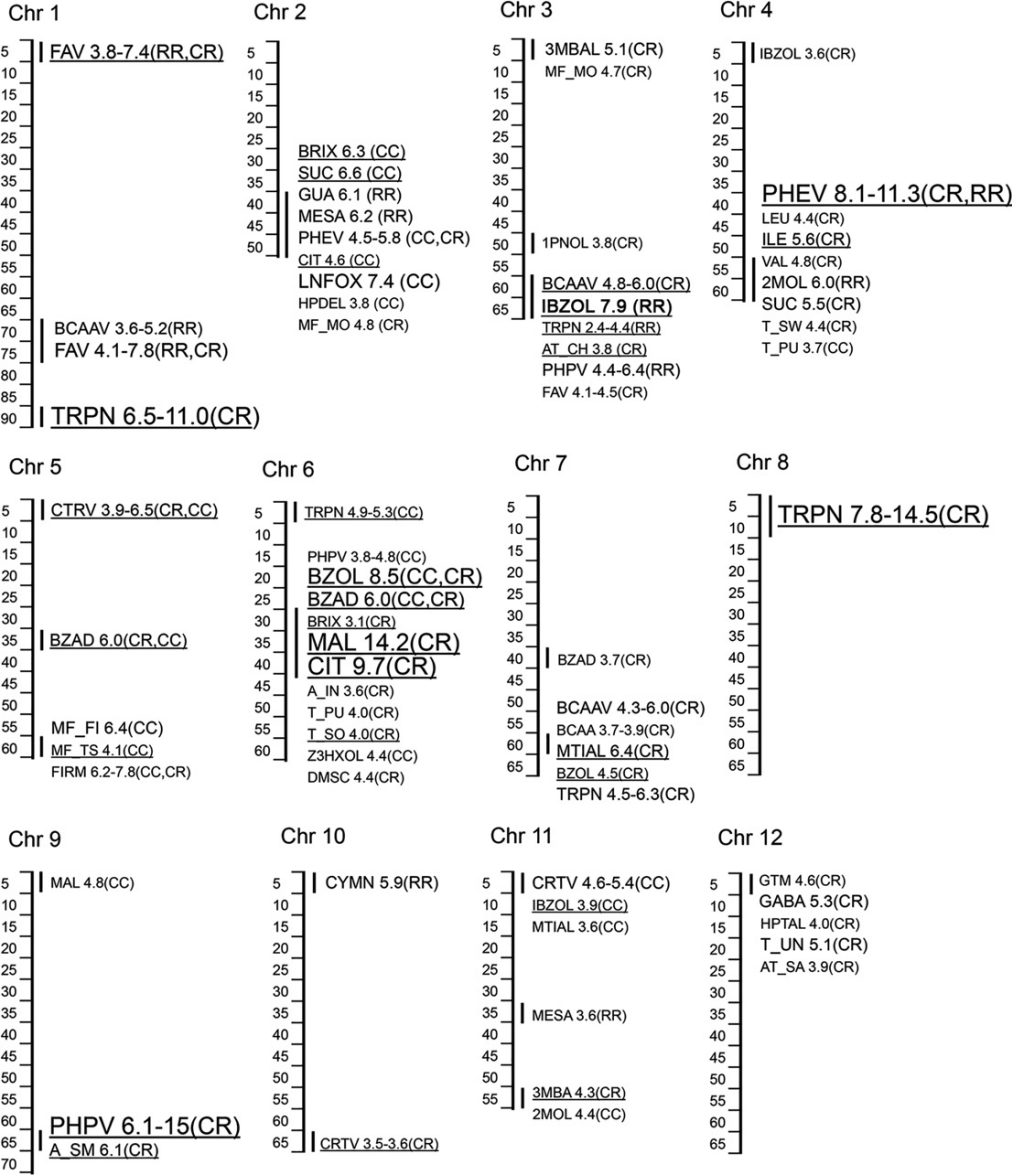
A physical map of tomato fruit flavor‐related quantitative trait loci (QTLs) detected in the F_6_ populations analyzed (QTLs detected in the F_2_ populations and the diversity panel are listed in Table S1). The scale of the map is in Mbp and the QTLs are binned every 5 Mbp. The font size of the traits is proportional to their Log *P* values, which are displayed on the right. In case of chemical group QTLs Min‐Max Log *P* are displayed. RR, CC, or CR indicate in what type of the cross round × round, cherry × cherry or cherry × round, respectively, a QTL was detected. The map is based on the F_6_ RIL data, but if a trait QTL was also observed in F_2_ or/and DP it is underlined in the map. Sensory traits: A_IN, aroma intensity; A_SM, smoky aroma; AT_CH, chemical aftertaste; AT_SA, salty aftertaste; MF_FI, firm mouthfeel; MF_MO, moist mouthfeel; MF_TS, tough‐skin mouthfeel; T_PU, pungent taste; T_SO, sour taste; T_SW, sweet taste; T_UN, unripe taste. Chemical traits: Fatty acid volatiles (FAV): *Z*‐3‐hexanol (Z3HXOL), *E*,*E*‐2,4‐heptadienal (HPDEL), 1‐pentanol (1PNOL); Terpene volatiles (TRPN): cymene (CYMN), linalool furanoxide (LNFOX); Carotenoid derived volatiles (CTRV): geranylacetone(GRON), damascenone (DMSC); Benzoate‐derived volatiles: benzaldehyde (BZAD), benzyl alcohol (BZOL); Branched‐chain amino acids (BCAA): leucine (LEU) and isoleucine (ILE), BCAA‐derived volatiles (BCAAV): 3‐methylbutanal (3MBAL), 2‐methylbutanol (2MOL), 2‐isobutylthiazole (IBZOL), 3‐methylbutanoic acid (3MBA); Methionine‐derived volatiles: methional (MTIAL); Phenylalanine‐derived volatiles (PHEV): 2‐phenylethanol (PHET), phenylecetaldehyde (PHAD), 1‐nitro‐2‐phenylethane (PHNE); Phenylpropanoid‐derived volatiles (PHPV): guaiacol (GUA), methyl salicylate (MESA), eugenol (EUG); refraction index (BRIX); sucrose (SUC); malic acid (MAL); citric acid (CIT); valine (VAL); glutamic acid (GTM); γ‐aminobutyric acid (GABA).

**Figure 3:**
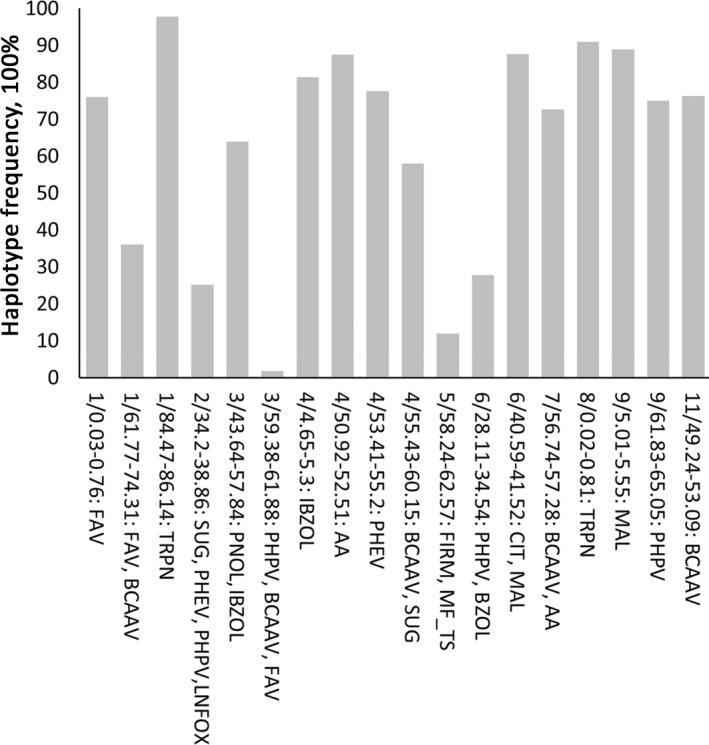
Percentage of representation of the half diallel parental haplotypes of 18 major quantitative trait locus (QTL) regions in the tomato genotypes that represent the genetic diversity of five breeding companies. The *x*‐axis legend represents chromosome/haplotype region, in Mbp, of the major trait QTLs in the respective haplotype region. The trait abbreviations are listed in the legend of Table 1.

A total of 239 marker‐trait associations (Log *P* > 3.5) were detected in the DP and in the segregating material (Table S1). Figure 2 summarizes the QTLs detected in the most advanced material: the F_6_ recombinant inbred lines (RILs). To estimate the representation of the parental haplotypes of the QTLs in the contemporary commercial tomato germplasm, the frequencies of haplotypes computed for 18 major QTLs detected in the half diallel were estimated in the collection of 1250 glasshouse tomato genotypes provided by five breeding companies (Figure 3; Table S2). Several loci harbored clusters of chemical compounds that have a common biochemical origin. Volatiles derived from fatty acids (FAVs) are the most representative and abundant group of volatiles in tomato fruit. Two significant QTLs for FAVs were detected at the top and at the bottom of chromosome 1 (Figure 2; Table S1). The haplotypes of the parents of the half diallel for these two QTLs were present in 76 and 35% of the breeders’ germplasm, respectively (Figure 3). Further towards the end of chromosome 1 another region, from 85.2 to 86.0 Mbp, harbored a strong QTL for five different volatile monoterpenes (TRPNs). Another locus strongly associated with multiple terpenes was found at the top of chromosome 8 (0.5–0.7 Mbp). Haplotypes of the half diallel parents of these two terpene QTLs had 98 and 90% representation in the breeders’ germplasm. The phenylpropanoid volatiles (PHPVs) and smoky fruit aroma (A_SM) were found to be associated with markers located between 64 and 65 Mbp on chromosome 9, and the strongest association was observed for the marker based on the polymorphism in the *NSGT1* gene, which has previously been established as the major factor controlling the release of these volatiles from tomato fruit (Tikunov *et al*., 2013). Each of the four half diallel parents had its own specific haplotype in this QTL region, which altogether were present in 75% of the breeders’ material. The variation in the sulfur‐containing amino acid derived volatiles methional (MTIAL) and 2‐isobutylthiazole (IBZOL) were modestly but significantly affected by a locus at about 5 Mbp on chromosome 11. IBZOL, which is partly derived from the branched‐chain amino acid (BCAA) catabolism, also showed a strong QTL (Log *P* = 7.98 and 29% of explained variance in the F_6_ RILs) on chromosome 3, in the region where QTLs for other BCAA volatiles were detected. The fruit content of the two intermediates of the TCA cycle, citric (CIT) and malic (MAL) acids, which are important for fruit acidity, revealed a strong QTL between 41.2 and 41.8 Mbp on chromosome 6, and 87% of the breeders’ genotypes contained the haplotype of half diallel parent R075. One of the strongest associations of biochemically related compounds was found between 51.8 and 56.8 Mbp on chromosome 4, which determined approximately 40% of the quantitative variation of the PHEVs 2‐penylethanol (PHET), phenylacetaldehyde (PHAD) and 1‐nitro‐2‐phenylethane (PHNE) – all products of catabolism of the amino acid phenylalanine. This strong association of PHEVs with the chromosome‐4 locus was observed across all the experiments: in the F_2_ and F_6_ populations, as well as in the DP. Of the contemporary breeding germplasm analysed, 78% was represented by the haplotypes of the half diallel parents (Figure 3; Table S2), and the frequency of the haplotypes associated with higher PHEV concentrations (haplotypes of C085 and C074 cherry parents) was 36% (Table S2). The median frequency of the half diallel haplotypes of the 18 QTL regions in the germplasm provided by the breeding companies was 75.5%.

### Sensory and metabolic analysis of near‐isogenic lines contrasting for the chromosome‐4 PHEV QTL region

**Figure 4:**
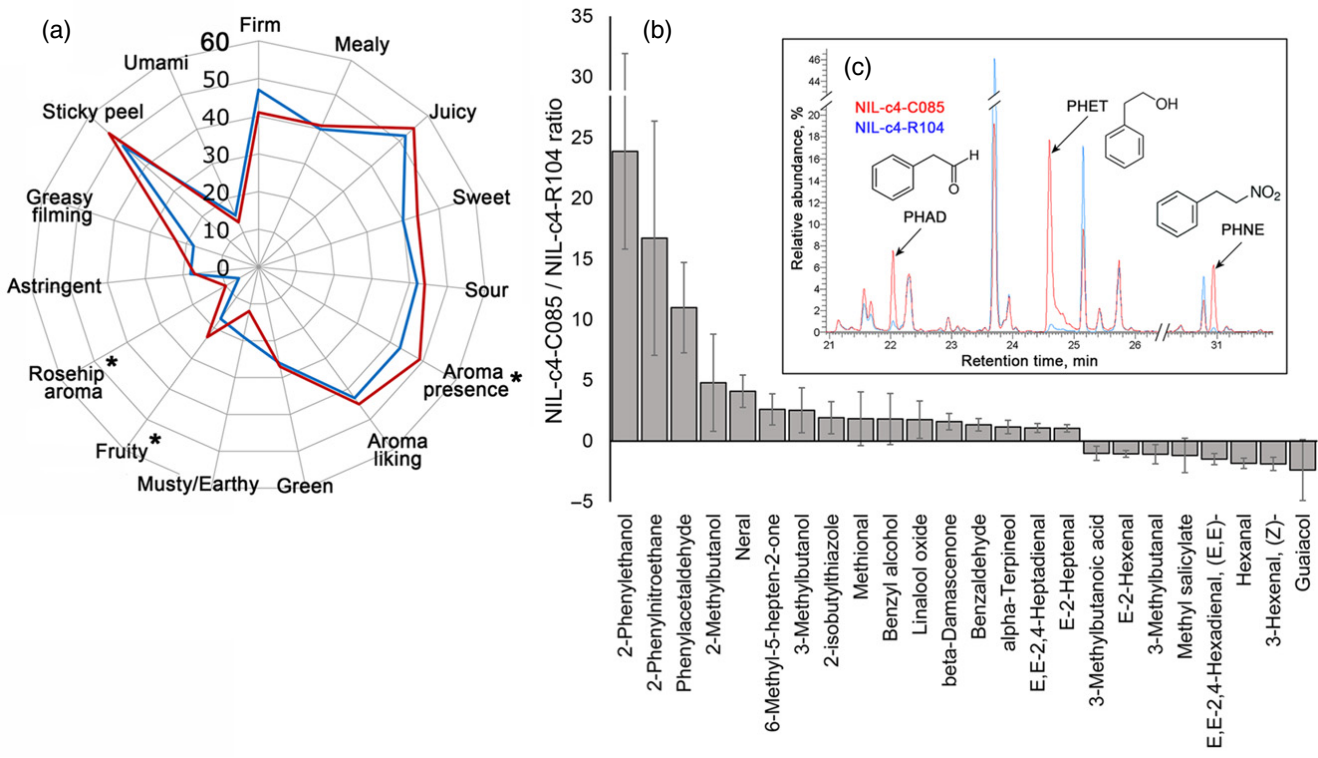
(a) Spider diagram showing the differences in sensory attributes between NIL‐c4‐C085 and NIL‐c4‐R104. (b) Bar chart showing fold difference in the content of aroma‐related volatile organic compounds (VOCs) in ripe fruits of NIL‐c4‐C085 compared with NIL‐c4‐R104. *Significance of the sensorial differences at *P* < 0.05.

The metabolic profiling and regression modelling of sensorial traits showed that the PHEVs showed a large quantitative variation in the fruits of our germplasm collection (Figures 1 and S3), and were positively correlated with sweet flavor (Table 1). In order to obtain more evidence whether variations in the content of PHET, PHAL and PHNE caused by the region on chromosome 4 could significantly affect the flavor of tomato fruits, two near‐isogenic lines (NILs), NIL‐c4‐C085 and NIL‐c4‐R104, were developed that carry either the C085 (high levels of PHEV) or R104 (low levels of PHEV) marker alleles in the associated locus on chromosome 4, between 52.66 and 57.16 Mbp, respectively. The flavor and volatile composition of the ripe fruits of the NILs were analysed by a trained sensory panel and by GC‐MS, respectively. The sensory panel found that NIL‐c4‐C085 had significantly (*P* < 0.05) higher scores for aroma presence, fruity aroma and rose‐hip aroma, and had 11, 17 and 24 times higher levels of PHAL, PHNE and PHET, respectively, compared with NIL‐c4‐R104 (Figure 4). The differences between these two NILs in the other important aroma volatiles was much lower. In addition, as sugars and organic acids are major determinants of tomato fruit flavor, we measured the content of glucose, fructose, sucrose, citrate and malate in the fruits of the NILs, and no significant difference was found for any of these compounds.

### Fine mapping of the PHEV chromosome‐4 locus

To narrow down the PHEV‐associated locus on chromosome 4 and to reduce the number of possible candidate genes, two successive rounds of recombinant plant selection were performed in a segregating F_2_ population of 5000, followed by a biochemical evaluation of selected F_3_ plants with a homozygous recombination in the PHEV QTL region (Figure S2). The two rounds of QTL fine mapping allowed us to map the PHEV QTL region to 330 kbp, from 54.52 to 54.85 Mbp, on chromosome 4 (Table S4). To validate and further narrow down the QTL region, a marker–trait co‐segregation analysis was performed with six F_3_ families segregating for specific regions in the QTL interval. For each family, up to 10 plants of each allelic class (homozygous for either the C085 or the R104 marker alleles in the recombined PHEV QTL region) were grown to maturity and ripe fruits were subjected to GC‐MS analysis. As a result, the PHEV‐associated region was narrowed down to 110 kbp, from 54.52 to 54.63 Mbp (Figure S4).

### Identification of a candidate gene underlying the PHEV QTL on chromosome 4

**Figure 5:**
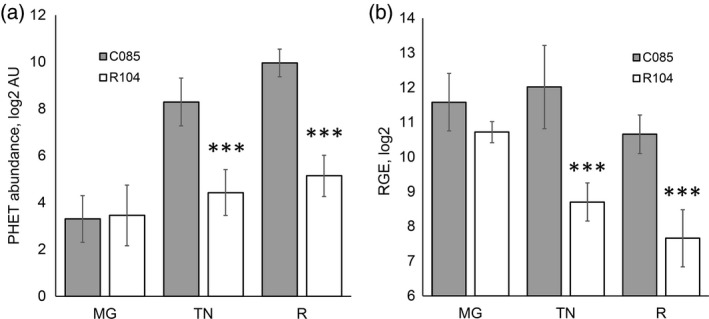
Comparative analysis of 2‐phenylethanol (PHET) abundance (a) and *FLORAL4* expression (b) in ripening fruits of F_3_ plants contrasting for the phenylalanine‐derived volatile (PHEV) chromosome 4 region: gray bars, plants with C085 marker alleles; white bars, plants with R104 marker alleles in the QTL region. Student’s *t*‐test for significant differences: ***P < 0.001.

Many aroma compounds accumulate during the ripening of tomato fruit through the transcriptional activation of genes of the corresponding biosynthetic pathways. To facilitate the identification of a candidate gene in the PHEV QTL region on chromosome 4, we studied the accumulation of PHEVs upon fruit ripening in plants contrasting for the PHEV QTL region. Fruits of six F_3_ plants of both allelic classes (C085 versus R104) were propagated in triplicate through cuttings and fruit material was analysed for volatile compounds at the mature green, turning (pink) and fully ripe stages. GC‐MS analysis revealed that the abundance of PHEVs increased upon ripening in fruits of both categories; however, the accumulation of these volatiles in fruits with the C085 allele was much stronger, resulting in an average 28‐fold difference between the two allelic classes in fully ripe fruits (Figure 5a).

According to the results described above, a candidate gene responsible for the differential accumulation of PHEVs in tomato fruit should satisfy the following criteria: (i) it must be located in the 110‐kbp PHEV‐associated interval on chromosome 4; (ii) it should have a higher expression of its C085 allele or the R104 allele should carry mutations leading to a functionally retarded R104 enzyme; and (iii) as the nitrogen‐containing PHNE is affected by the QTL in the same way as PHET and PHAD, it is expected, according to the current model of PHEV biosynthesis in tomato (Tieman *et al*., 2006), that the candidate enzyme is likely to act upstream of phenylethylamine, the common precursor of these three volatiles. Phenylethylamine can be produced by decarboxylation of the amino acid phenylalanine mediated through the action of a decarboxylase. Eleven genes have been predicted in the region according to the tomato genome annotation (The Tomato Genome Consortium, 2012) (Table S5). Among them, a protein encoded by *Solyc04g063350*, located at 54.58 Mbp (SL2.40 tomato genome release), showed a high similarity to 3‐methyl‐2‐oxobutanoate dehydrogenase, an enzyme that is part of the branched‐chain amino acid dehydrogenase complex involved in the catabolism of amino acids. The 3‐methyl‐2‐oxobutanoate dehydrogenase forms the E1 subunit of the complex and possesses decarboxylase activity. As *Solyc04g063350* was selected as a candidate for the regulation of phenolic volatiles with floral odor characteristics, and is located on chromosome 4, it was denoted as *FLORAL4*. cDNA sequences of *FLORAL4* were cloned from C085 and R104 and revealed no nucleotide polymorphisms leading to deleterious mutations (Figure S5). Gene expression analysis of *FLORAL4* in ripening fruits of the aforementioned F_3_ plants revealed a much higher transcript abundance in fruits with the C085 allele versus fruits with the R104 allele at all ripening stages, fully in line with the results of the volatile analyses (Figure 5b).

### CRISPR‐CAS9 mediated mutagenesis of *FLORAL4*

**Figure 6:**
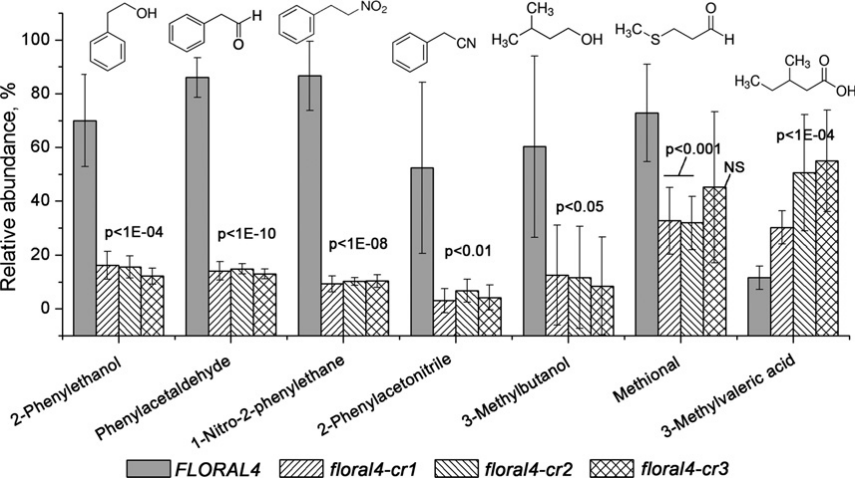
Relative abundance of phenylalanine‐derived (PHEV) and other amino acid‐derived volatiles significantly affected in fruits of the three CRISPR‐CAS9 mutants *floral4‐cr1, floral‐cr2* and *floral‐cr3*, compared with wild type fruits (*FLORAL4*). Bars represent the average abundances of the volatiles (*n* = 5 plants per genotype) relative to the maximum across all individual replicate plants. Significance: Student’s *t*‐test *P* value; NS, non‐significant.

One of the RILs (4‐131) developed from the C085 × R104 crossing population, which carries the homozygous C085 allele of *FLORAL4* and has a high abundance of PHEVs (Figure S3), was used to mutagenize *FLORAL4* using CRISPR‐CAS9‐mediated gene editing. This resulted in three independent mutants carrying different mutant alleles, called *floral4‐cr1*, *floral4‐cr2* and *floral4‐cr3*. *Floral‐cr1* carried two in‐frame deletions of three and six nucleotides in the first and the second guide sequences, respectively (Figure S6). *Floral‐cr2* had a large 65‐bp deletion between the guide sequences, which resulted in an open reading frame shift and a premature stop codon. The same non‐sense effect was found in *floral4‐cr3*, which carried a three‐nucleotide deletion and a one‐nucleotide insertion. All the mutant plants were phenotypically indistinguishable from the wild‐type plants. Ripe fruits of five homozygous T_1_ plants per mutant were analysed for VOC composition using GC‐MS. This analysis revealed that all three mutants showed a similar, between five‐ and nine‐fold reduction of the three PHEVs in their ripe fruits compared with non‐edited wild‐type (WT) plants (Figure 6). Interestingly, another structurally related phenolic nitrogen‐containing volatile, 2‐phenylacetonitrile, which is also produced from Phe in plants (Miki and Asano, 2014), but to our knowledge has not been regarded so far as an important aroma compound in the dedicated literature, also showed a strong reduction in the mutant fruits. 3‐Methylbutanol, which is produced from the BCAA leucine or a related keto‐acid (Kochevenko *et al*., 2012), showed a consistent significant reduction (by approximately four‐fold) in the three mutants compared with the control. The abundance of methional, which is derived from methionine (Di *et al*., 2003), was also reduced in the mutant fruits. On the other hand, the mutations in *FLORAL4* caused a moderate increase in 3‐methylvaleric acid content. Other volatiles showed an occasional quantitative difference between one of the mutants and the WT, but these differences were much smaller and not significant (Table S6). Similar to PHET and 3‐methylbutanol, the contents of glycoconjugate forms of these volatiles were significantly reduced in the fruits of *floral4* mutants, whereas several flavonoid and phenylpropanoid metabolites showed increased abundances in the mutant fruits (Table S7). An analysis of primary metabolites revealed that ripe fruits of all three *floral4* mutants had a significantly higher content of the BCAA leucine, approximately 0.125 ± 0.04 mg g^–1^ FW (fresh weight), compared with WT fruits (approx. 0.032 ± 0.01 mg g^–1^ FW) (Figure S9a; Table S7). Phe content did not significantly differ between the WT and the mutants (approx. 1.37 ± 0.2 mg g^–1^ FW), however, and neither did other primary metabolites (Figure S9a, Table S7). To check for possible off‐target mutants the two guide sequences used for CRISPR‐CAS9 mutagenesis were searched in the tomato genome using blast. *Solyc03g118420* appeared to be the only gene predicted in the tomato genome that had a 100% similarity of the first 15 bp of the 23‐bp sgRNA1 guide, and therefore could be potentially targeted by CRISPR‐CAS9. We sequenced the coding region of this gene in all three mutants and did not observe any mutations compared with the sequence of the WT RIL4‐131. An alternative strategy was applied to support the conclusions made based on the results of the CRISPR‐CAS9 mutagenesis using virus‐induced gene silencing (VIGS) of *FLORAL4* in detached fruits of a different commercial variety Solarino (Appendix S1). The results of this transient silencing were in agreement with the results of the CRISPR‐CAS9 mutagenesis: a reduction in the expression of FLORAL4 and a reduction in PHEV, BCAAV and methional content was observed in the silenced fruits (Figure S10).

## DISCUSSION

The multivariate analysis of the chemical and sensorial variation showed that the tomato fruits tested in this study were spread along a major gradient ranging between sweet, spicy, fresh and typical tomato flavors, which have an intuitively positive perception, at one end, and a rather negative earthy and unripe taste, with mealy texture, at the other end. Thus, it is not surprising that sweet taste, which has been evolved in plants to indicate the ripeness of fruit to potential seed dispersers, has unripe tomato taste among its counterparts. The higher expressions of sweet, spicy and typical tomato flavor coincided with the higher content of chemicals that typically accumulate in tomato fruit during ripening: sugars, citric acid and volatile compounds derived from the amino acid phenylalanine (PHEV) and branched‐chain amino acids (BCAAV). The regression modeling of the sensory attributes suggests a few major scenarios for the sensory–chemical associations: (i) a specific flavor characteristic is exclusively associated with one or a few biochemically related chemicals; (ii) a flavor characteristic is associated with many, often even biochemically unrelated, chemicals; (iii) several distinct flavor characteristics can be affected by a single chemical. For instance, variation in the smoky aroma was exclusively associated with PHPV. Indeed, the aroma of guaiacol is often described as smoky and the smell of eugenol might evoke a memory of a dental office, as eugenol is commonly used in dentistry (Robin *et al*., 1999; Sarrami *et al*., 2002). In addition to this exclusive control of the smoky flavor, PHPV also had high positive correlation for the other sensory attributes that potentially negatively affect flavor quality, such as unripe and earthy taste, bitter, chemical and rough aftertaste, and a negative correlation for the sweet, tomato, spicy and fresh attributes. This might either result from a direct sensorial effect or a ‘fly in the ointment’ effect, when the perception of one strong unpleasant component can compromise or mask the experience of all the positive components, even though sufficient contents of these are present. The sweet, spicy, tomato flavor models appeared to be more complex and involved compounds from different chemical classes, with positive contributions (sugars, citrate, PHEVs, BCAAVs and FAVs) and negative contributions (PHPVs, CRTVs and some terpenes). According to the RF regression analysis, sugars and PHEVs are both interchangeably important for sweet taste and aroma; however, the importance of 2‐phenylethanol (PHET) for the sweet aroma model noticeably increases compared with the importance of sugars. Many different chemicals showed a considerable statistical importance for explain the sensorial variation. Compared with chemicals important for flavor, fewer flavor attributes could be associated with significant QTLs. This discrepancy may reflect the high complexity of sensorial perception: many different chemicals may participate to produce a certain sensorial expression, and each of these chemicals may, in turn, be affected by multiple genetic loci. Therefore, the total genetic impact on the variation of a sensorial trait may be divided over many genetically and functionally independent loci. In addition, sensorial perception is prone to a high degree of subjectivity, leading to substantial variation among the judges and experimental ‘noise’. This also limits the detection of associated sensorial QTLs and makes a detailed chemical analysis of paramount importance for the understanding of the genetic regulation of flavor. Nevertheless, some co‐associations of chemical and sensorial traits could be observed. For instance, the smoky aroma, which is exclusively determined by phenylpropanoid volatiles, showed one of the most significant associations with chromosome 9. Sweet taste revealed a strong association with chromosome 4 and co‐located with the chemicals that showed a positive contribution to the model of this sensory trait: PHEVs and sucrose. Sour taste co‐located with a major QTL for the organic acids citrate and malate on chromosome 6. The sensory–chemical associations discovered in this study, using RF regression analysis, have a statistical nature and their causality must be proven with a dedicated sensorial analysis of specifically developed fruit material. Some of such associations have already been shown to be causal (Baldwin *et al*., 2008; Tikunov *et al*., 2013). In this study, a sensorial analysis of NILs developed to differ in PHEV content showed significantly higher levels of the fruity, rose‐hip aroma of tomato fruits, which accumulated higher quantities of PHET, PHAD and PHNE. This suggests that variation in PHEV content could affect the expression of these sensorial characteristics. The effect of PHEV on tomato flavor has been reported in several other studies. In combination with sugars and acids, PHET showed a significant positive effect on the expression of the tropical and fruity aroma of tomato fruit (Baldwin *et al*., 2008). On the other hand, a very high concentration of PHAD (approx. 0.25 µg g^–1^ FW) was suggested to be the reason for the unpleasant perception of the fruits produced by tomato plants cv. M82 carrying an introgression (the so‐called *malodorous* locus) from the wild tomato *Solanum pennellii* in chromosome 8 (Tadmor *et al*., 2002). In our material – the fruits of F_6_ RILs that carried the high PHEV *FLORAL4* allele – the concentration of PHAD was on average 0.02 µg g^–1^ FW (with a maximum of about 0.05 µg g^–1^ FW) (Figure S3). This is about 10 times lower than in the fruits that carried *malodorous*, but still above the reported odor threshold of 0.004 µg g^–^^1^ FW (Baldwin *et al*., 2000b). The level of PHET in some RILs was above 1 µg g^–1^ FW, which is the reported odor threshold for PHET (Baldwin *et al*., 2000b). These odor thresholds, which must be considered with care as they were determined by measuring odor activity in water solutions, suggest that both PHET and PHAD content in our fruit material are at concentrations perceivable by humans. We cannot exclude a combined sensorial effect, however, as these volatiles exhibit a similar type of sweet, floral and fruity odor. A gene encoding an aromatic amino acid decarboxylase (AADC1) was suggested to be the key factor responsible for the effect of the *malodorous* locus (Tieman *et al*., 2006). To our knowledge, however, the M82 × *S. pennellii* was the only population in which this locus has been detected and is unlikely to be common in cultivated tomato. No QTLs for PHEVs were found on chromosome 8 in the tomato material used in the present study. On the other hand, a strong association of PHEVs with the 50–60 Mbp region on chromosome 4 was detected across all experiments, starting from the DP of 94 commercial cultivars to more advanced RIL tomato material. Other studies, conducted using different tomato material, have also reported QTLs for PHEVs in the same region on chromosome 4 using specific crossing populations of *S. lycopersicum* (Saliba‐Colombani *et al*., 2001; Zanor *et al*., 2009) or different genome‐wide association studies (GWAS) panels (Bauchet *et al*., 2017; Tieman *et al*., 2017), which suggests that this locus may have a widespread effect across the entire cultivated tomato germplasm. The analysis of the haplotype frequencies in this QTL region showed that up to 78% (36% carrying the high PHEV allele) of the breeders’ germplasm was represented by three haplotypes of the parents of the populations made in this study. Out of 18 major QTL regions, only five were represented with a frequency of <40% by the diallel haplotypes in the representative sample of the genetic variation of five breeding companies, and 11 showed a frequency over 70%. This makes the validation of the QTLs detected in this study worthwhile for practical breeding as a considerable proportion of the genetic variation, which underlies these QTLs, is already present in the contemporary breeding material.

According to the available annotation and to the protein similarity analysis (Figures S7 and S8), FLORAL4 did not belong to the true aromatic amino acid decarboxylase family, such as AADC (Tieman *et al*., 2006), but to the mitochondrial 2‐oxoisovalerate dehydrogenase/decarboxylase family that acts in the catabolism of BCAAs, as the E1 subunit of the BCKDC complex. In plants, this complex catalyzes the second step of BCAA catabolism: the decarboxylation of the BCAA deamination products (Mooney *et al*., 2002). To our knowledge there have been no studies reporting the involvement of this class of enzymes in the catabolism of aromatic amino acids. Our reverse‐genetics analyses, however, demonstrated that the introduction of deleterious mutations in *FLORAL4* by gene editing led to a severe depletion of volatiles derived from phenylalanine in the mutant fruits. A less strong, but significant reduction was also observed for 3‐methylbutanol. At the same time, leucine, the amino acid precursor of 3‐metylbutanol, showed a significant four‐fold increase in fruits of the *FLORAL4* mutants. In contrast to leucine, no increase in phenylalanine content was observed in the mutant fruits. Besides the PHEVs, phenylalanine serves as a precursor for thousands of phenolic compounds that play important structural and physiological roles in plants. The existence of complex mechanisms involved in the maintenance of phenylalanine homeostasis in plants has been demonstrated recently (Yoo *et al*., 2013; Qian *et al*., 2019). Interestingly, although the PHEV content was reduced in the mutant fruits, a significant enrichment of some flavonoids and phenylpropanoids was observed, which in this case could hypothetically keep phenylalanine content in equilibrium. *In vitro* enzyme assays could help to resolve the activity properties of FLORAL4; however, our attempt to study the activity of FLORAL4 in a bacterial overexpression system with various substrates, such as phenylalanine, leucine or their deamination products (keto‐acids), using the AADC *Escherichia* *coli* assay (Tieman *et al*., 2006), did not yield any products, as was determined by non‐targeted GC‐MS and LC‐MS profiling. Therefore, despite the obvious *in planta* effect, the decarboxylase activity of FLORAL4 remains putative until a dedicated functional assay is developed and the decarboxylase activity has been demonstrated unequivocally.

## EXPERIMENTAL PROCEDURES

### Diversity panel of 94 commercial hybrids

The DP, previously reported upon by Tikunov *et al*. (2005) and Van Berloo *et al.,* (2008), consisted of 94 tomato (*S. lycopersicum*) genotypes, mainly commercial glasshouse F_1_ hybrids obtained from six different tomato seed companies, each with its own breeding program. As such, the cultivars were selected to represent a considerable collection of genetic and therefore phenotypic variation, not just between tomato types (18 cherry, 55 round and 21 beef), but also within the individuals of each type. This study was deliberately performed blind. For classification, breeders generally use a combination of: (i) fruit diameter and (ii) number of locules in the fruit (fl). For fl, the criteria were as follows: cherry‐type fl, 2; round fl, 3; beef fl, 4 or more. All cultivars of the association panel as well as all the following trials were grown under standard glasshouse conditions at a single location in Wageningen, the Netherlands. Nine plants, randomly distributed over three adjacent glasshouse compartments, were grown for each cultivar. Pink‐staged tomato fruits of all plants were picked on two consecutive days. To mimic the conditions from the farm to the fork, fruits were stored for 1 week at 15°C and then changed to 20°C for 24 h prior to analysis (taste trials and/or freezing for metabolic analysis). During this period, the fruits continued to ripen and, at the moment of sampling, the fruits were fully red ripe, resembling the conditions at the time of consumption. For each cultivar, a selection of red ripe fruits (12 for round and beef tomatoes and 18 for cherry tomatoes) was pooled to make a representative fruit sample. The fruit material of the DP as well as all other fruit material used in this study was immediately frozen in liquid nitrogen, ground in an analytical electric mill (IKA 2900001; IKA, https://www.ika.com) and stored at −80°C before analyses.

### F_2_ segregating populations

Based on the phenotyping results of the DP, four different cultivars, denoted as R104 and R075 (medium‐sized round fruit) and C085 and C074 (cherry fruit), were selected as representing a large part of the variation for most of the sensorial characteristics and metabolic traits. A half diallel was developed using one parent of each of these four cultivars. Each parent was selected after predicting phenotypic values for all parental inbred lines using QTL models developed from the trait association analysis of the DP, as described previously (Van Berloo *et al*., 2008). The half diallel cross resulted in six segregating populations: one cherry × cherry, one round × round and four cherry × round populations (Figure S1d). Experiments with F_2_ fruits were carried out in two consecutive trials. In the first trial, 300 F_2_ plants (50 plants were grown per population) were grown to maturity and ripe fruits (between six and 15 fruits per plant) were harvested. Fruit material was sampled and stocked as described above for the association panel. The fruit material of this trial was analysed for flavor‐related primary metabolites (sugars, organic acids and amino acids) and volatile aroma compounds. Based on the multivariate analysis of metabolic data from the first trial, 96 plants (approx. 16 plants per population) were selected to represent maximum variation of the metabolic traits and propagated by making nine cuttings per plant. The cuttings were grown in the second trial in a randomized block design and ripe fruits of each set of nine plants were harvested and subjected to a sensorial evaluation by a trained sensory panel.

### F_6_ recombinant inbred lines

Three of the six segregating populations, R104 × C085 (cherry × round), C085 × C074 (cherry × cherry) and R104 × R075 (round × round), were developed further into F_6_ RIL populations. One hundred F_6_ RILs per population were analysed in three consecutive trials. RILs were grown under standard glasshouse conditions using a randomized block design with nine plants per RIL and three plants per block. Ripe fruits were harvested, sampled and analysed as described above for the DP and the F_2_ populations. Four independent F_6_ trials were performed: one with 300 RILs (100 of each population) and three trials with 100 RILs (50 per population) of CC and CR populations (Table S11). For the R104 × C085 RILs the 50 RILs with the largest fruits were selected to minimize the possible fruit‐size contrast.

### Development of PHEV near‐isogenic lines

Four F_6_ RILs from population 4 (C85 × R104) have been selected that were heterozygous in the chromosome‐4 PHEV QTL region and were almost completely homozygous for the rest of the genome. Up to 20 F_7_ plants per RIL were grown and genotyped for zygosity in the PHEV locus. Fruits of two F_8_ NILs contrasting in the PHEV locus were harvested at the fully ripe stage and used for both sensory and VOC analysis.

### Sensory analysis

To determine the sensory profiles of the tomatoes a sensory panel was trained based on the Quantitative Descriptive Analysis (QDA®) method (Stone *et al*., 2004). The panel (10 people, with a mean age of 49.1 ± 7.8 years) was trained to distinguish and score (on a scale from 0 to 100) the sensory attributes described in Table S9 using training subsets of tomato cultivars. The panelists evaluated tomato fruits of 17 tomato genotypes per day. The same cultivar was always used as a reference throughout the tasting sessions for the same sensorial experiment. Data acquisition and analysis were performed with fizz (Biosystemes, https://www.biosystemes.com) and an analysis of variance (ANOVA) in spss 15.0 (IBM, www.ibm.com), to test for significant differences between the fruit samples of different genotypes (as factor products and panelists; as interaction product × panelist). The sensorial analysis was performed on fruit material of the DP of 94 cultivars for three independent trials. Sensorial analysis of fruits of the F_2_ segregating populations was performed during one trial on the plant material, multiplied as described in the F_2_ population development section above. Sensory evaluation of the F_6_ RILs was carried out with 50 RILs of C074 × C085 (cherry × cherry) and 50 RILs of R104 × C085 (round × cherry) RIL populations.

Fruits of the NILs contrasting for the PHEV locus were tasted by 19 assessors. Before tasting, all assessors participated in flavor training sessions in which the flavor attributes based on the research of Verkerke *et al.,* (1998) and definitions of Meilgaard (2016) were explained, discussed and adjusted for fresh tomatoes in this research, resulting in the sensory descriptors shown in Table S10. For the flavor test, fruits of the same ripeness were selected (based on color and firmness) to standardize each sample as far as possible. Each assessor tasted six fruit pieces originating from different fruits of each of the NILs. The fruit samples were coded and presented in a randomized sequence, in a neutral environment. The statistical analysis was carried out by means of an anova with Student’s *t*‐test *post‐hoc* analysis (*P* < 0.05). Sensory analysis was performed at Wageningen UR Greenhouse Horticulture (Bleiswijk, the Netherlands) and at Food & Biobased Research (Wageningen, the Netherlands).

### Metabolic analyses

Volatile compounds were quantified and identified using headspace solid‐phase micro‐extraction–gas chromatography–mass spectrometry (SPME‐GC‐MS), as described by Tikunov *et al*. (2005).

Soluble solid content (SSC) and acidity of the fruit material were analysed using a portable handheld PAL BX/ACID3 analyzer (Atago, https://www.atago.net). Frozen fruit powder was thawed at 22ºC room temperature, centrifuged and the juice was subjected to analyses according the analyzer manual.

Individual sugars, and organic and amino acids, were analysed using an Agilent 6890 gas chromatograph (https://www.agilent.com) coupled to a Pegasus III time‐of‐flight (TOF) mass spectrometer (LECO, https://www.leco.com) using the sample preparation and instrumental methods described previously by Osorio *et al*. (2012) and Carreno‐Quintero *et al*. (2012), respectively.

Semi‐polar secondary metabolites were analysed using the LTQ Orbitrap LC‐MS system composed of a HyPurity^TM^ C18 column (ThermoFisher Scientific, https://www.thermofisher.com), an Acquity UPLC coupled to a photodiode array (PDA) detector (both from Waters, https://www.waters.com) and an LTQ/Orbitrap hybrid mass spectrometer (ThermoFisher Scientific), as previously described by van der Hooft *et al*. (2012).

### Metabolomic data analysis

Both volatile and primary metabolite GC‐MS raw data were processed by metalign freeware (https://www.wur.nl/en/show/MetAlign‐1.htm). The compound quantitative profiles and mass spectra were extracted using msclust freeware (https://www.wur.nl/en/show/MetAlign‐1.htm; Tikunov *et al*., 2012), and the compounds were annotated by matching the extracted mass spectra using the National Institute of Standards and Technology (nist) ms search software (https://chemdata.nist.gov/mass‐spc/ms‐search/) with the main nist mass spectra library (https://www.nist.gov/srd) for volatile compounds and the golm metabolome database (http://gmd.mpimp‐golm.mpg.de) for primary metabolites and using retention indices. Tentative identification was assigned to mass spectral matches of >700 with a retention index deviation of <20. The identities of all the volatiles, sugars, and organic and amino acids reported in the QTL analysis were confirmed by using authentic chemical standards.

### Genotyping

Genomic DNA was extracted from young leaves of the individual plants using the cetyl trimethylammonium bromide (CTAB) method (Steward and Via, 1993; Kabelka *et al*., 2002). Genotyping was performed using a 5510 SNP Infinium array (Table S8), as described in (Víquez‐Zamora *et al*., 2013). For the genotyping of the fine‐mapping population, KASP™ assays were designed for single‐nucleotide polymorphisms (SNPs) selected in the target QTL region on chromosome 4.

### QTL analysis

#### Association analysis of the diversity panel

Association mapping of the DP was performed by regression, with correction for fruit type (cherry versus round) using the following model: *y* = type + marker + error, where ‘*y*’ is a vector of trait values, ‘type’ is a vector to indicate the tomato type (cherry or round/beef), and ‘marker’ is a vector of the SNP marker score (scores take values 0, 1 and 2). Marker effects were tested with a two‐sided Student’s *t*‐test.

#### QTL analysis in F_2_ and F_6_ populations

A QTL analysis was performed for all traits measured on the F_2_ population, consisting of a half diallel of four parents (six crosses), and on the F_6_ populations, consisting of three crosses. In an initial step the phenotypic analysis of each experiment was carried out and genotypic means were obtained for each experiment. QTL analysis of the F_2_ population was performed per trait, simultaneously for all crosses. We used a mixed model that corrected for the genetic background as well as for the *rin* mutation, which was present in crosses with parent R075: *y* = cross.genotype + rin + marker + error, where random terms are shown underlined. Vector ‘*y*’ contains the values of a trait. Random term ‘cross.genotype’ corrects for the genetic background and allows for a genetic variance component for each cross. The fixed term ‘rin’ corrects both for additive effects and for dominance of the *rin* gene in crosses involving parent R075, which carries the *rin* mutation: rin = rin_crosses.(cross + rin_effects + cross.rin_effects), where rin_crosses is an indicator vector that equals ‘1’ for crosses with R075 as a parent and ‘0’ otherwise. Rin_effects is a two‐column matrix containing the additive effects and dominance effects of *rin*, respectively. The fixed term ‘marker’ is a four‐column matrix, where each element *ij* contains the number of alleles in genotype *i* that were inherited from parent *j*. The fixed marker terms were tested by a Wald statistic and corresponding parental effects, standard errors of parental effects and *P* values were obtained.

For the F_6_ populations, traits were measured in plants of crosses C085 × R104 (CR), R104 × R075 (RR) and C085 × C074 (CC). Only genotypes of the F_2_ RR population that were not homozygous for the mutant allele of the *rin* gene were selfed to the F_5_ generation. Only F_5_ plants homozygous for the WT *rin* locus were selected to produce F_6_ RILs. QTL analysis of the F_6_ RIL population was performed individually for each cross, because the three crosses were very heterogeneous. A simple regression model was used to test marker–trait associations: *y* = marker + error. The null hypothesis that the marker effect is equal to zero was tested with a two‐sided Student’s *t*‐test. All analyses were performed in genstat (VSNi, https://www.vsni.co.uk/software/genstat), unless otherwise noted.

### Representation of QTL haplotypes in the commercial tomato germplasm

To study how well the haplotypes of the QTLs detected in the half diallel were represented in the commercial tomato germplasm, six international breeding companies have anonymously provided DNA for a total of 1250 tomato accessions used in their breeding programs. In total, 199 KASP™ markers were developed to cover 18 major QTL regions (Table S3). The DNA samples of a total of 1250 tomato genotypes were provided by five breeding companies and the KASP™ assays were performed by VHL Genetics (https://www.vhlgenetics.com). After filtering out poorly performing markers (153 remained) and accessions (variable depending on a marker set), haplotypes were computed for the 18 QTL regions.

### Fine‐mapping population and fine mapping of the phenolic VOC QTLs

Two RILs contrasting for most of the flavor‐associated QTLs, further denoted as RIL4‐066 and RIL4‐128, were crossed to develop a segregating population of 5000 individuals used for the fine mapping described in this study (the complete fine‐mapping scheme is described in Figure S2).

### CRISPR‐CAS9 editing of *FLORAL4*

A CRISPR‐CAS9 construct to mutagenize *FLORAL4* was designed in the binary transformation vector pAGM4723 using golden gate assembly (Engler *et al*., 2009). Two specific guide RNAs were designed for mutagenizing *FLORAL4* and were expressed under the control of the AtU6p small RNA promoter. To design specific single‐guide RNAs (sgRNAs), an alignment of *FLORAL4* and its orthologs was made and two unique guide sequences downstream of PAM(‐NGG) sites were identified and designed: sgRNA1, 5′‐CCTTGGAACAGGTTGCCAATCAA‐3′ (593–615 bp of *FLORAL4*), and sgRNA2, 5′‐CCTGCCACTATGGTTCTAATGAG‐3′ (619–641 bp of *FLORAL4*). Tomato cotyledons were transformed with *Agrobacterium tumefaciens* AGL1 harboring the CRISPR‐CAS9 construct, according to the protocol described by Ellul *et al*. (2003). Plants of the T_1_ generation were screened for homozygous mutations by sequencing the *FLORAL4* gene, and ripe fruits of these homozygous mutants (five plants per mutant) and the corresponding WT plants were analyzed by GC‐MS for volatile compounds.

### Comparative statistical analyses, regression modeling, sequence analyses and visualization

The Student’s *t*‐test in excel (Microsoft, https://www.microsoft.com) was used to compute the significance of differences in chemical composition in this study. Multivariate comparative analyses and their visualization were performed using principal components analysis, implemented in past 3 freeware (https://folk.uio.no/ohammer/past/). Random forest (RF) regression analysis of the individual sensory attributes from the metabolic profiling data was performed in r using the randomforest function (https://cran.r‐project.org/web/packages/randomForest/randomForest.pdf). Metabolic profiling data of the DP, F_2_ and F_6_ data sets (*n* = 273) were 2log transformed. To remove the batch effect of different trials, quantitative profiles of individual compounds were scaled by a trial mean. In the randomforest function, an importance value of each compound in the regression model was quantified by the increase in the mean square error (MSE) after permuting (500 times) the values of that compound for the out‐of‐bag samples, standardized by the standard deviation of the difference. The positive or negative directionality of the effects of the chemicals in the RF models was determined using Pearson correlation.

DNA and protein sequence analyses were performed and visualized in clc genomic workbench 12 (https://digitalinsights.qiagen.com).

### Real‐time quantitative PCR

Quantitative PCR was performed on the *FLORAL4* (*Solyc04g063350*, solgenomics.net) transcript in an ABI 7500 Fast Real‐Time PCR system (Applied Biosystems, now ThermoFisher Scientific). RNA was extracted from tomato fruit powder of eight F_3_ tomato lines from three different ripening stages: mature green, turning and ripe. Primers used for amplification of the target gene were designed using primer 3 plus (http://www.bioinformatics.nl/cgi‐bin/primer3plus/primer3plus.cgi): *FLORAL4* Fw, 5′‐GTATCGACCCGTTGAGGAAA‐3′; *FLORAL4* Rv, 5′‐CACCTTCTCTGCTGCTTGAA‐3′. The RT‐PCR mixture (duplo) consisted of: 22 μl 2× iQ SYBR Green super mix (Bio‐Rad, https://www.bio‐rad.com), 11 μl ultrapure deionized water, 4.4 μl forward primer (3 μm), 4.4 μl reverse primer (3 μm) and finally 2.2 μl cDNA (10 ng/μl) for each sample to get a final volume of 44 μl. The housekeeping gene *β‐actin* was used as reference gene for quantification. The relative gene expression for each sample was calculated using the cycle‐threshold (*C*_t_) values of the gene of interest and the reference gene *β‐actin*.

## MATERIAL AVAILABILITY

Seeds of the F_6_ RIL populations are freely available from the authors, upon request.

## CONFLICT OF INTEREST

The authors declare no conflicts of interest.

## AUTHORS CONTRIBUTIONS

FMD, JM, YT, IC, FCM, MNdV, CL, WV, RR and AvH. performed the experiments. JP, FvE, YT, RF, CM, AvH, FvE, RR and AB analysed the data. YT, RR, JP, CL, RV and AB wrote the article.

## Supporting information


**Figure S1.** Principal components analysis of the genetic variation of the diversity panel of 94 tomato cultivars based on the 5510 SNP array used in the study.
**Figure S2.** Fine‐mapping process of the PHEV QTL on chromosome 4.
**Figure S3.** The abundances of PHET and PHAD in the fruits of the F_6_ RILs.
**Figure S4.** Comparative analysis of the abundance of PHEVs in the six recombinant F_3_ families, which segregate for C085 (high PHEVs) and R104 (low PHEVs) marker alleles in the recombined region on chromosome 4.
**Figure S5.** Protein alignment of FLORAL4 cloned from two parental genotypes of the segregating populations.
**Figure S6.** Partial DNA alignment of *floral4* CRISPR‐CAS9 mutants compared with the *FLORAL4* wild‐type sequence of C085 (high PHEVs).
**Figure S7.** Dendrogram created based on a protein alignment of different amino acid decarboxylases retrieved from the National Center for Biotechnology Information (NCBI) reference protein database.
**Figure S8.** Protein alignment of plant amino acid decaroxylases retrieved from the National Center for Biotechnology Information (NCBI) reference protein database.
**Figure S9.** Leucine (A) and phenylalanine (B) content (mg g^–1^ FW) in ripe fruits of the FLORAL4 Crispr‐Cas9 mutants and the wild‐type fruits (WT).
**Figure S10.** FLORAL4 expression (A) and volatile relative abundance (B) in fruits of cv. Solarino where FLORAL4 was silenced using virus‐induced gene silencing (VIGS), compared with GUS control fruits.

**Table S1.** Significant QTLs detected in the tomato diversity panel, and F_2_ and F_6_ populations.
**Table S2.** Representation of the QTL haplotypes in the breeders’ commercial tomato germplasm.
**Table S3.** SNP markers and their physical position used to make haplotypes of the 18 important QTL regions and asses their representation in the breeders’ germplasm.
**Table S4.** Results of a Kruskal–Wallis marker/PHET association after the fine mapping of the PHEV trait on chromosome 4.
**Table S5.** Genes predicted by the tomato genome annotation in the fine‐mapped PHEV‐associated region on chromosome 4.
**Table S6.** Relative abundances of the VOCs identified in the CRISPR‐CAS9 *floral4* mutant fruits.
**Table S7.** Quantitative differences in primary polar and secondary semi‐polar non‐volatile compounds between ripe fruits of the FLORAL4 Crisp‐Cas9 mutants and the wild type (WT) fruits.
**Table S8.** Sequences of the SNP marker of the tomato Infinium SNP array.
**Table S9.** Plant amino acid decarboxylase proteins.
**Table S10.** Sensory attributes used by the sensory panels.
**Table S11.** Overview of types of quantitative data generated per population, and number of experiments per data type.

**Appendix S1.** Virus‐induced gene silencing (VIGS) of FLORAL4 in detached tomato fruit.

## Data Availability

All relevant data can be found within the manuscript and its supporting materials.
