# Supplementary material for: The genetic and functional analysis of flavor in commercial tomato: the FLORAL4 gene underlies a QTL for floral aroma volatiles in tomato fruit
Source: Plant J. 2020 Jun 21;103(3):1189–204. doi: 10.1111/tpj.14795 (PMC7496274; doi:10.1111/tpj.14795)
Supplement: Supplementary file 1 — Figure S1. Principal components analysis of the genetic variation of the diversity panel of 94 tomato cultivars based on the 5510 SNP array used in the study. Figure S2. Fine‐mapping process of the PHEV QTL on chromosome 4. Figure S3. The abundances of PHET and PHAD in the fruits of the F6 RILs. Figure S4. Comparative analysis of the abundance of PHEVs in the six recombinant F3 families, which segregate for C085 (high PHEVs) and R104 (low PHEVs) marker alleles in the recombined region on chromosome 4. Figure S5. Protein alignment of FLORAL4 cloned from two parental genotypes of the segregating populations. Figure S6. Partial DNA alignment of floral4 CRISPR‐CAS9 mutants compared with the FLORAL4 wild‐type sequence of C085 (high PHEVs). Figure S7. Dendrogram created based on a protein alignment of different amino acid decarboxylases retrieved from the National Center for Biotechnology Information (NCBI) reference protein database. Figure S8. Protein alignment of plant amino acid decaroxylases retrieved from the National Center for Biotechnology Information (NCBI) reference protein database. Figure S9. Leucine (A) and phenylalanine (B) content (mg g–1 FW) in ripe fruits of the FLORAL4 Crispr‐Cas9 mutants and the wild‐type fruits (WT). Figure S10. FLORAL4 expression (A) and volatile relative abundance (B) in fruits of cv. Solarino where FLORAL4 was silenced using virus‐induced gene silencing (VIGS), compared with GUS control fruits. [file TPJ-103-1189-s001.pdf]

Supporting figures

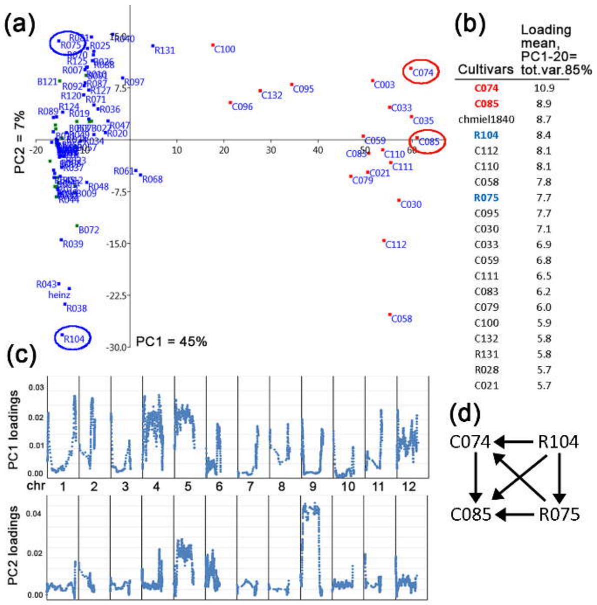

Supporting Figure S1. Principal Components Analysis of the genetic variation of the diversity panel of 94 tomato cultivars based on the 5510 SNP array used in the study. (a) Scores plot showing the genetic variation along the first two principal components. A half-diallel cross was made using the parents of four cultivars (encircled) representative for the genetic, chemical and sensorial variation in this panel. This cross included two cherry (R074 and R085) and two round genotypes (R075 and R104). (b) Average score of top 20 cultivars in the first 20 principal components of the PCA, which explain a total of 85% genetic variation between the cultivars. (c) Loadings of the SNP markers in the first two principal components. (d) The half-diallel crossing scheme, the arrows show the crosses made and arrowheads indicate female parent of each of crosses.

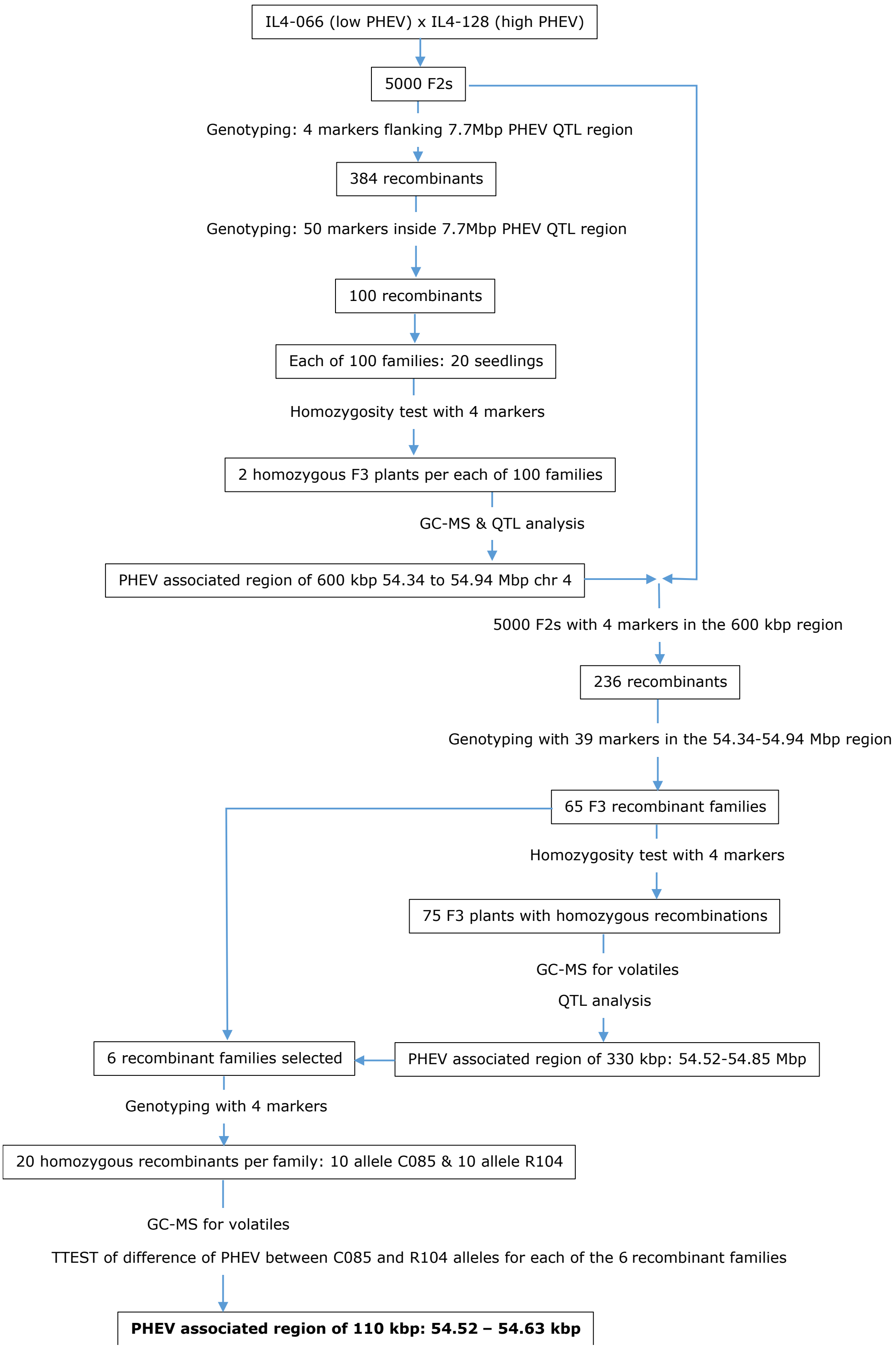

Supporting Figure S2. Fine mapping process of the PHEV QTL on chromosome 4. Two rounds of recombinant selection were performed with the entire population of 5000 plants. In the first round, the 5000 plants of the population were genotyped with four SNP markers spanning the 7.7 Mbp (from 53.0 to 60.7 Mbp) of the segregating PHEV region in chromosome 4. 384 recombinants were further genotyped with 50 SNP markers inside the 7.7 Mbp region to select a set of 100 F2 plants with recombinations spanning the entire PHEV region as uniform as possible. For each of the 100 selected recombinants twenty F3 seedlings were grown and two plants out of twenty with a homozygous recombination in the QTL region were selected with four SNP markers.

Ripe fruits of these homozygous plants were harvested, sampled and analysed for VOC by GC-MS. In the second round, the recombinant search was repeated using the 5000 segregating F2 plants to find additional recombinants in the PHEV associated region yielding 236 recombinants. These recombinants were genotyped with 39 markers. 75 selected F3 plants homozygous for the recombined PHEV locus were subjected to genotypic and VOC analyses. A Kruskal-Wallis test was performed to estimate marker-trait association at each of the two rounds. As a final step, sets of 20 plants were selected from each of 6 F3 recombinant families, carrying either a homozygous C085 or a homozygous R104 allele in the recombined regions. Ripe fruits of these plants were analysed for VOC by GC-MS and Student's t-tests were performed for PHEV content in each of the 6 families to finally define the boundaries of the PHEV associated region.

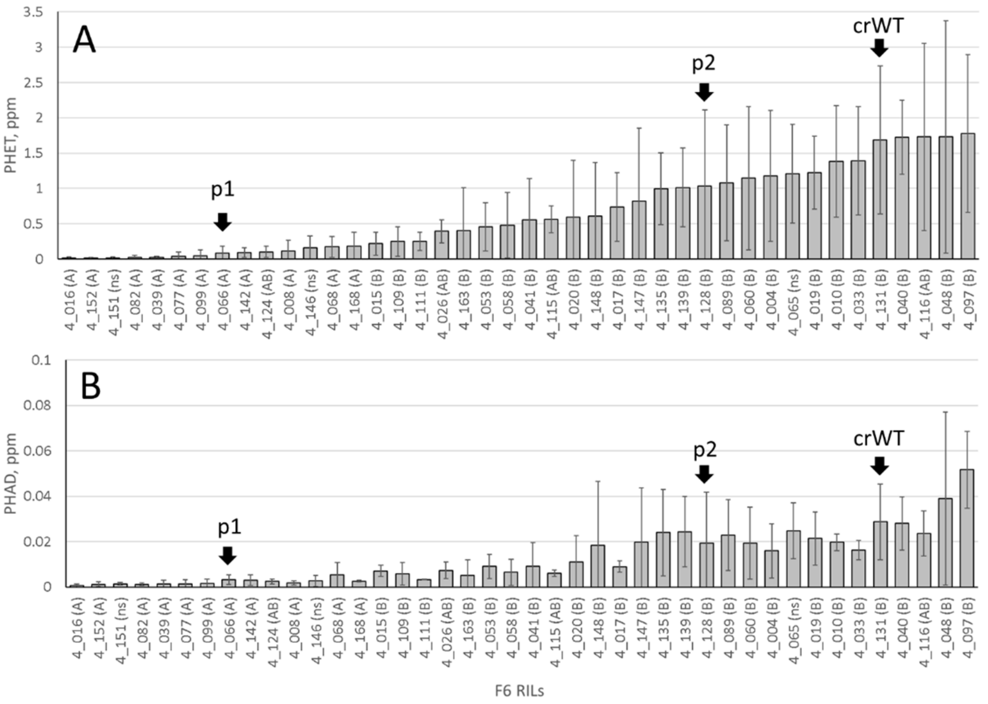

Supporting Figure S3. The abundance (ppm) of PHET and PHAD in the fruits of the F6 RILs. A, B or AB on the right of the RIL IDs correspond to R104, C085 or heterozygous alleles of an Infinium array SNP marker rs8242 (the closest to *FLORAL4*). The two parental RILs 4_066 and 4_128 used to generate the fine mapping F2 population are marked as p1 and p2, respectively. crWT is RIL 4_131 used as wild type recipient for the *FLORAL4* CRISPR-CAS9 mediated mutagenesis.

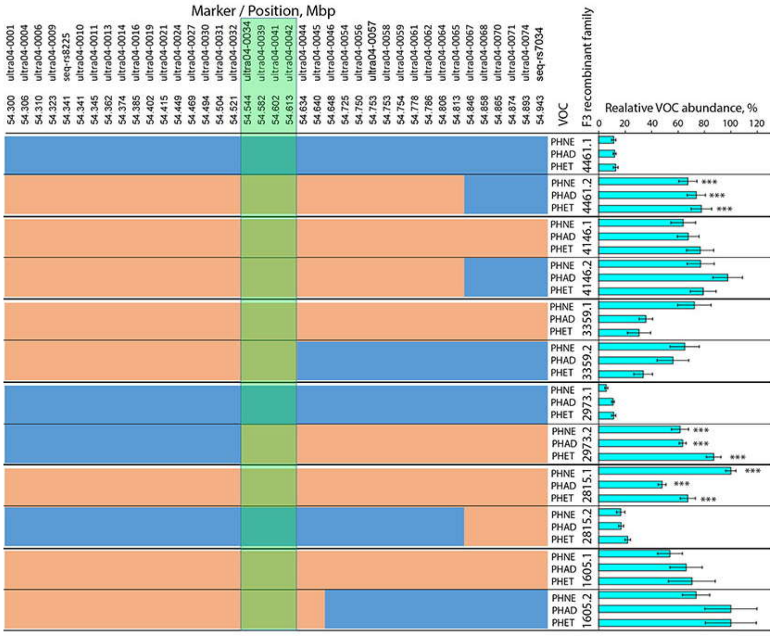

Supporting Figure S4. Comparative analysis of PHEV abundance in the six recombinant F3 families which segregate for C085 (high PHEV) and R104 (low PHEV) marker alleles in the recombined region on chromosome 4. The significant difference in PHEV abundance, in particular, between the segregants of family 2973 and the absence of difference in a family 3359 indicate that the PHEV associated region is located between markers ultra04-0032 at 54.521 Mbp and ultra04-0044 at 54.634 Mbp. ***Student's t-test p-value <0.001.

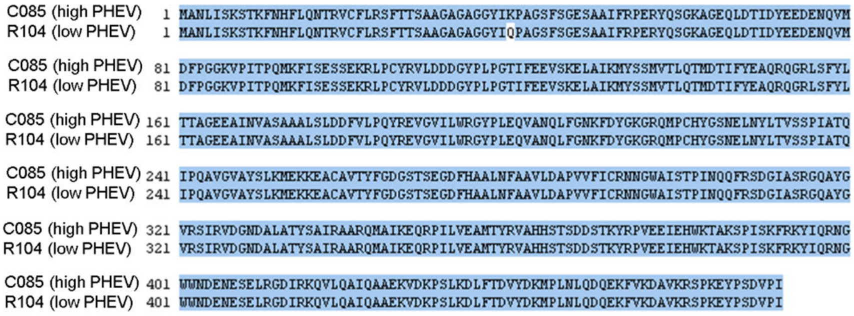

Supporting Figure S5. Protein alignment of FLORAL4 cloned from two parental genotypes of the segregating populations: C085 which has higher abundance of PHEV in ripe fruits compared to R104.

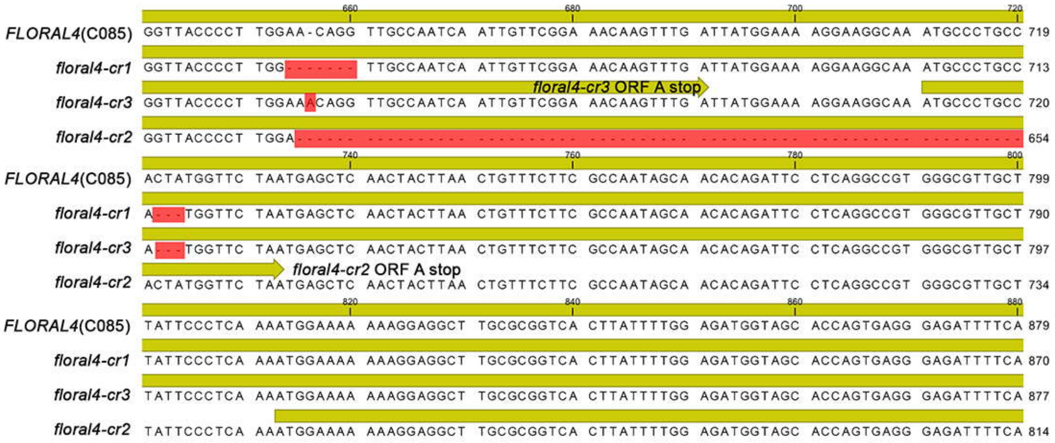

Supporting Figure S6. Partial DNA alignment of *floral4* CRISPR-CAS9 mutants compared to the *FLORAL4* wild type sequence of C085 (high PHEV). The gene region from 640 to 814 bp of the CDS is displayed and the positions of the two guide RNAs used are boxed. The mutations are highlighted in red: *floral4-cr1* – a deletion of 6 bp at position 655 of the CDS within the sgRNA1 region and a 3 bp deletion at position 722 of the CDS within the sgRNA2 region; *floral4-cr2* – a deletion of 65 bp between the two guides; *floral4-cr3* – insertion of an A residue at position 656 of the CDS in the sgRNA1 region and a 3 bp deletion at position 722 of the CDS in the sgRNA2 region. The mutations in *floral4-cr2* and *-cr3* both lead to premature stop codons.

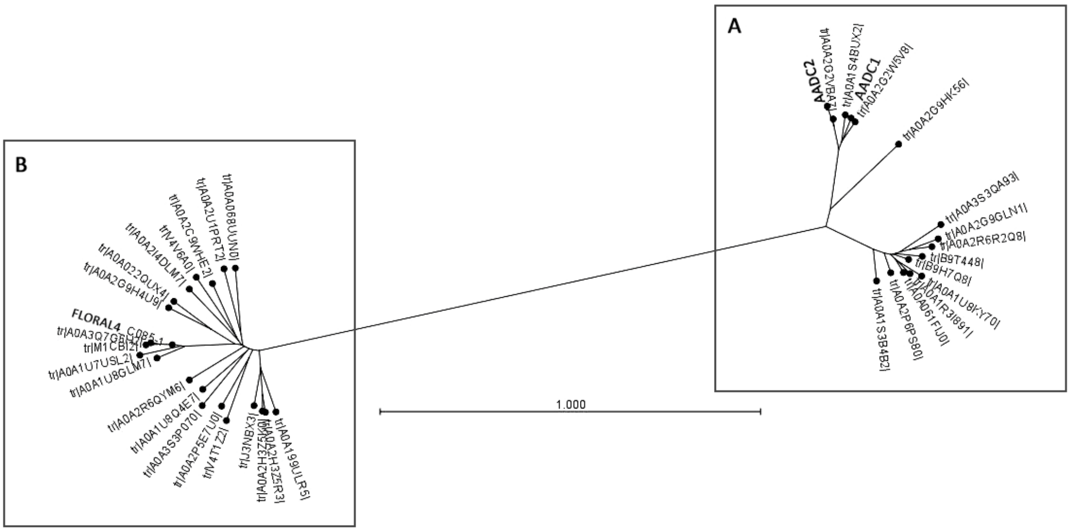

Supporting Figure S7. Dendrogram (Neighbor Joining, Jukes-Cantor distance) created based on a protein alignment of different amino acid decarboxylases retrieved from the NCBI reference protein database. The annotation of the protein sequences used is described in **Supporting Table S9**. Cluster A consists of aromatic amino acid decarboxylases, including AADC1 and AADC2 of the tomato malodorous locus (Tieman et al., 2006), serine, histidine and glutamate decarboxylases. Cluster B consists of proteins annotated as 2-oxoisovalerate decarboxilases, E1 subunits of the BCKDC complex and FLORAL4 of the present study.

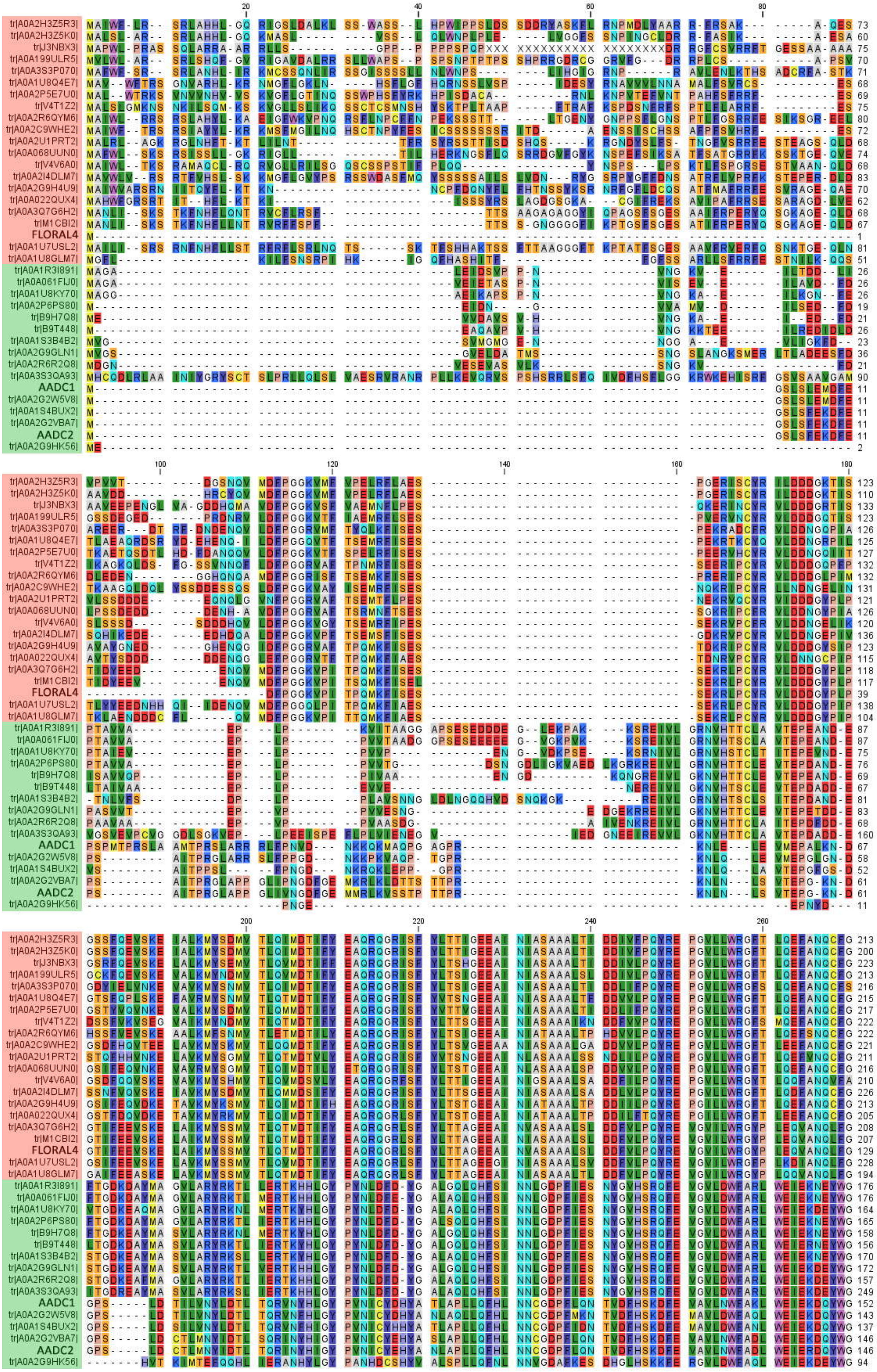

Supporting Figure S8. Continued on the next page

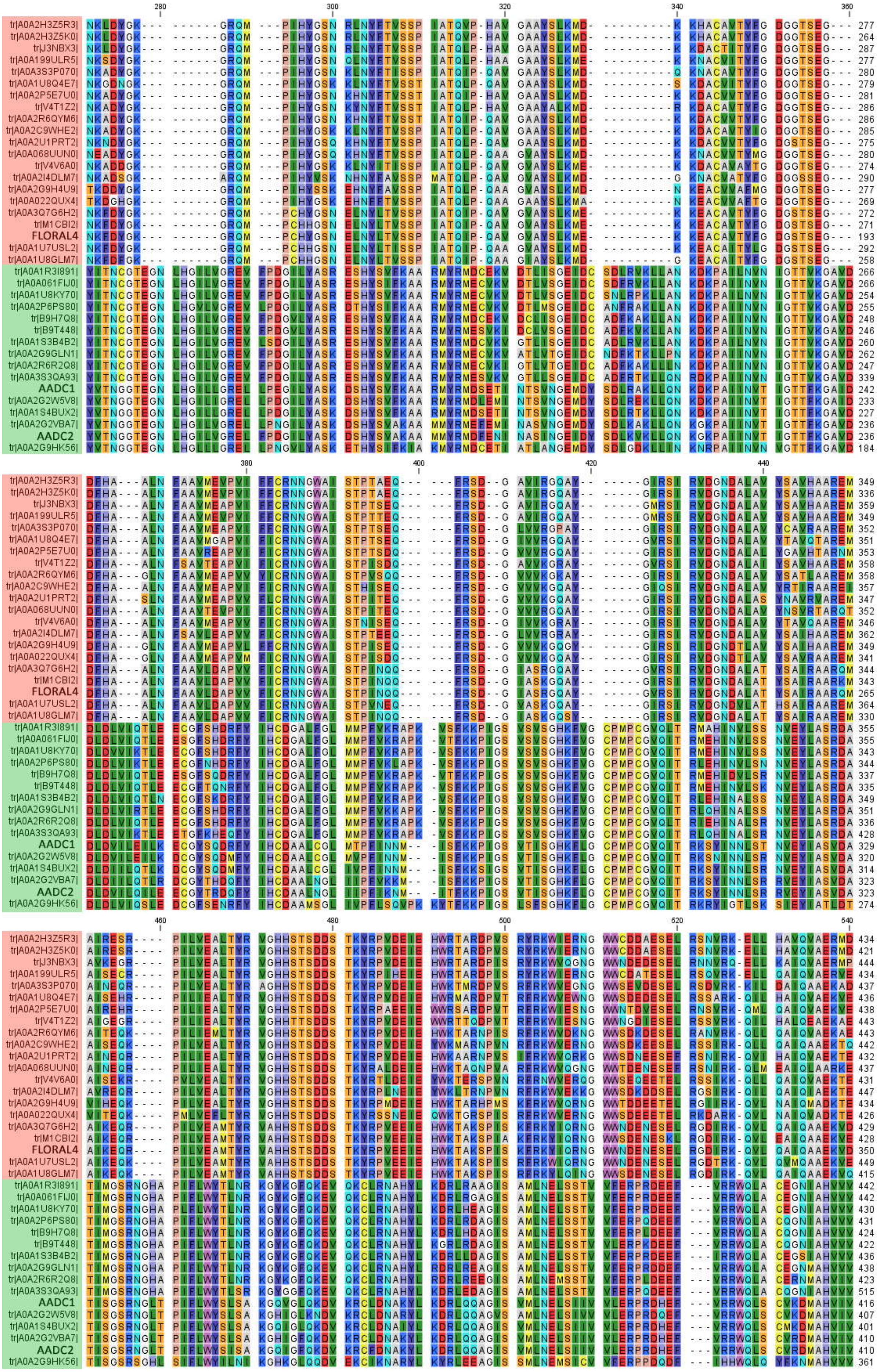

Supporting Figure S8. Continued on the next page

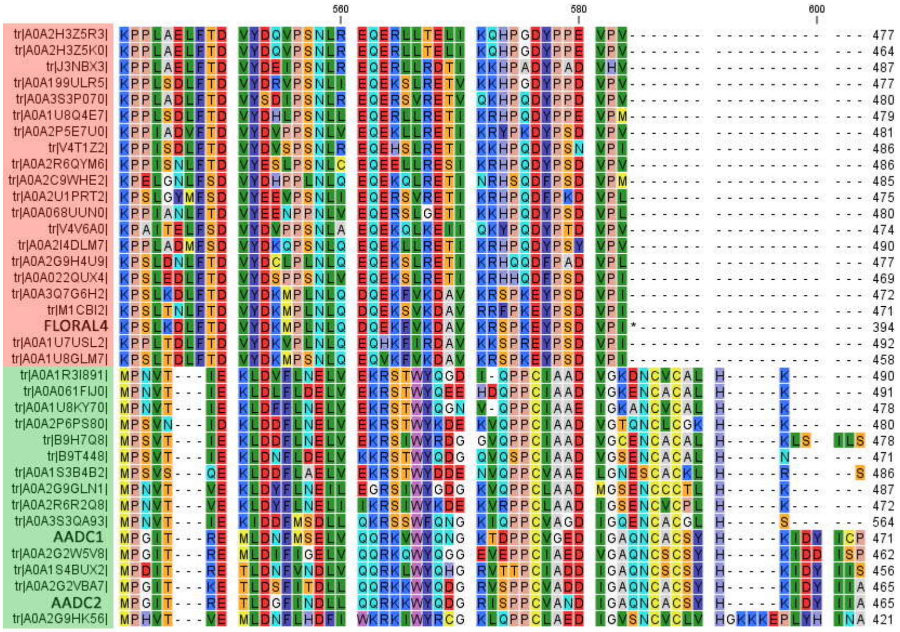

Supporting Figure S8. Protein alignment of plant amino acid decaroxylases retrieved from the NCBI reference protein database. The annotation of the protein sequences used is described in **Supporting Table S9.** IDs of the proteins of clusters A and B (in Supporting Figure S7) are highlighted in green and red, respectively.

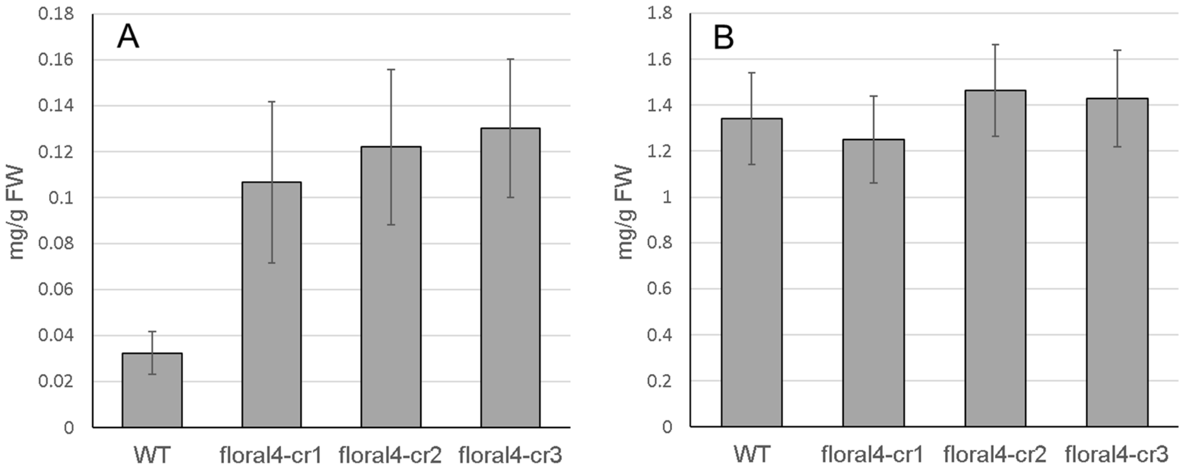

Supporting Figure S9. Leucine (A) and phenylalanine (B) content (mg/g FW) in ripe fruits of the *floral4* Crispr-Cas9 mutants and the wild type fruits (WT).

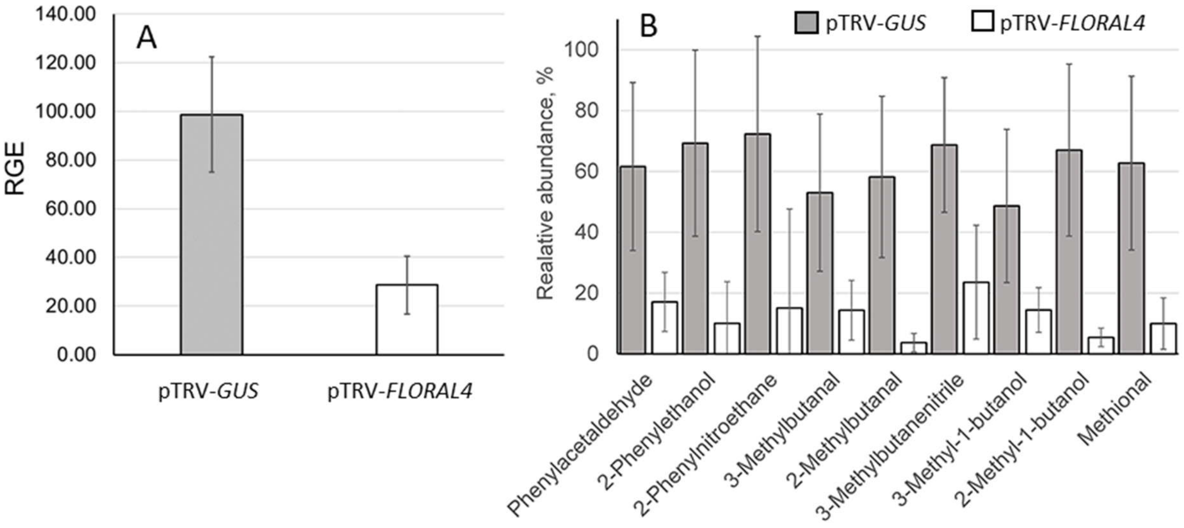

Supporting Figure S10. *FLORAL4* expression (A) and volatile relative abundance (B) in fruits of cv. Solarino where *FLORAL4* was silenced (n=20) using VIGS compared to GUS control fruits (n=20).
