# Supplementary material for: The genetic and functional analysis of flavor in commercial tomato: the FLORAL4 gene underlies a QTL for floral aroma volatiles in tomato fruit
Source: Plant J. 2020 Jun 21;103(3):1189–204. doi: 10.1111/tpj.14795 (PMC7496274; doi:10.1111/tpj.14795)
Supplement: Supplementary file 3 — Appendix S1. Virus‐induced gene silencing (VIGS) of FLORAL4 in detached tomato fruit. [file TPJ-103-1189-s003.docx]

**Supporting Materials and Methods S1.** Virus induced gene silencing of FLORAL4 in detached tomato fruit

For virus induced gene silencing, mature green tomato fruits of a mini plum *Solanum lycopersicum* L. cv. Solarino (Rijk Zwaan B.V.) were used. After plasmid verification and gene expression analysis, *A. tumefaciens* strains GV3101 containing either pTRV2-*FLORAL4* or pTRV2-*GUS* as a negative control were used for virus induced gene silencing. The A. tumefaciens strains containing the vectors were grown at 28°C (200 rpm) in YEB medium containing the appropriate antibiotics. After two days, GV3101 cultures were transferred into 50 ml tubes containing culture medium (YEB medium, 200 mM acetosyringone, 50 mg/mL kanamycin and 1M MES). The next day, GV3101 overnight cultures were recovered by centrifugation for 8 min at 4000 rpm. Next, the pellet was re-suspended into MMA-induction medium (2 g sucrose, 0.5 g MS salt without vitamins, 1 mL 1M MES, 1 mL 200 mM acetosyringone, pH 5.6), wrapped in aluminum foil and incubated for 3-6 hours at room temperature. For tomato fruit infiltration, all GV3101 strains containing pTRV2-*FLORAL4* or pTRV2-*GUS* were combined with pTRV1 in a 1:1 ratio just before infiltration in the green house. Agro-infiltration was performed into the stylar apex of the detached tomato fruit using a 1 mL syringe with a 0.4 mm x 20 mm needle. After infiltration, the tomato fruits were incubated under controlled conditions at 22°C and 70% RH in the greenhouse. Both pTRV2-*GUS* and pTRV2-*FLORAL4* fruits were harvested after two weeks when they reached turning-pink stage, which was comparable for both pTRV2-*GUS* and pTRV2-*FLORAL4*. Fruit were then processed and subjected to gene expression and volatile compound analyses to study the effect of VIGS as described in the Materials and Methods of the main text.
